# Tuning Self-Trapped Exciton Emission in 1D White-Light Emitting Perovskites Through Halide Composition and Synthesis Route

**DOI:** 10.1021/acsomega.5c01452

**Published:** 2025-06-09

**Authors:** Bar Bader, Elisheva Michman, Ioannis N. Gkikas, Ioannis Spanopoulos, Ido Hadar

**Affiliations:** † Institute of Chemistry, The Center for Nanoscience and Nanotechnology, and Casali Center for Applied Chemistry, 26742 The Hebrew University of Jerusalem, Jerusalem 9190401, Israel; ‡ Department of Chemistry, 7831 University of South Florida, Tampa, Florida 33620-5250, United States

## Abstract

Low-dimensional halide perovskites (LDHPs) are promising candidates for optoelectronic applications, specifically light emission. LDHP’s reduced-dimensional frameworks allow exceptional tunability of the optoelectronic properties. Self-trapped exciton emission (STE) is an intriguing characteristic of some LDHPs, offering a compelling mechanism for white-light emission. In this work, we study the effect of halide substitution and synthetic methods on the optical properties, with a particular focus on STE emission, of one-dimensional LDHPs using (2,5-dmpz)­Pb­(Br_*x*_Cl_1–*x*_)_4_ (dmpz = dimethylammonium piperazine) as a model system, exhibiting high emission quantum efficiency and structural consistency. Mechanochemical approaches, including manual grinding and ball milling, were employed to synthesize pure and mixed halide compounds, enabling precise composition control. Structural characterization revealed that ball milling produces materials with improved crystallinity and a homogeneous halide distribution. Optical characterization showed an anticorrelation between the absorption onset and STE emission energies, with STE properties influenced by both halide composition and a synthesis route. Moreover, we found that the STE emission of the mixed halide compounds evolved with time. The post-synthesis evolution of the STE emission spectrum is associated with local lattice distortions rather than long-range structural changes. The emission quantum efficiency of these compounds was measured and reached a value of 35%, among the highest values measured for 1D broad emitters. These findings provide additional insights into the design of next-generation LDHP compounds exhibiting STE emission and development of white-light-emitting devices based on them.

## Graphical abstract

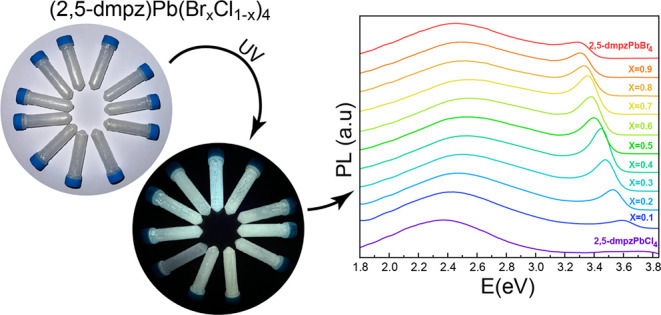

## Introduction

As technology advances, the demand for high-performance and cost-effective materials continues to grow, driving innovation in various fields. Metal halide perovskites (MHPs) are a novel family of semiconductor materials with a soft crystal lattice that have arisen in the recent decade as candidates for optoelectronic applications, such as solar cells,^1–7^ light emitting devices (LEDs),^8–12^ and radiation detectors.^13–17^ MHPs combine simple and scalable solution processing, with exceptional, tunable electronic properties, leading to extensive research of these compounds, both fundamental and applicative.^18–20^ In general, MHPs are composed of a 3D network of corner-sharing metal-halide octahedra (MX_6_, M—Pb^2+^ or Sn^2+^, X—I^–^, Br^–^, or Cl^–^) supported by a small cation (methylammonium, formamidinium, or Cs^+^), forming the stoichiometry of AMX_3_.^13,21,22^

Low-dimensional halide perovskites (LDHPs) are compounds that maintain the same octahedral base-unit as in the 3D MHPs but modify the connectivity to form lower dimensionality (2D, 1D, or 0D). LDHPs allow additional connectivity motifs, such as edge- and face-sharing.^23,24^ These compounds usually form by the introduction of a bulkier cation instead of or in addition to the small A cation.^25–31^ LDHPs combine the desired characteristics of MHPs with improved stability.^32–34^ Specifically, LDHP compounds are increasingly recognized as promising light-emitting materials, offering unique advantages over their three-dimensional counterparts.^35–38^ In addition to their superior stability, they enable additional tunability of the optical properties,^39–42^ greater structural diversity,^43–45^ efficient exciton radiative recombination,^46,47^ and improved light emission yield.^48–50^ A unique property of some LDHP compounds is the emission of a broad, white-light spectrum in addition to or instead of the narrow band-edge emission, which is typical for standard semiconductors and is associated with free-excitons (FEs).^51–64^ These white-light-emitting low-dimensional halide perovskite (WLE-LDHP) derivatives can address specific challenges in the field of solid-state lighting devices and white LEDs by simplifying the emission process and hence have drawn much attention in recent years. The possibility of realizing a single component white-light emitter bears significant advantages in comparison to traditional phosphor-based systems, which require balancing the luminescence intensity of multiple components and managing mutual absorption between different phosphors, often resulting in suboptimal light output and color rendering.^65–68^ Such single component white-light-emitting devices may reduce production costs and enhance the efficiency of simple illumination devices.

The broad emission of WLE-LDHPs is associated with the self-trapped exciton (STE) mechanism.^39,69–71^ The formation of STE states stems from the reduced dimensionality of WLE-LDHPs, which promotes strong exciton–phonon coupling within the soft lattice. This coupling can lead to modifications in the local potential field and the formation of excited state trap levels with lower energies.^39,72^ The radiative recombination of these STEs produces emission within the visible region, characterized by a broad spectral range and a significant Stokes shift.^47,73^ While the existence of STE emission in many WLE-LDHPs is well established, the exact mechanism is still not well resolved. Moreover, the realization of electrically driven white-light-emitting devices based on WLE-LDHPs has not been achieved to date. Hence, an in-depth study of the STE luminescence mechanism is a critical necessity for the integration of WLE-LDHPs in next-generation illumination applications.

Recent studies have thus concentrated on understanding and controlling the fundamental processes involved in STE formation and emission.^74–79^ Investigating lattice distortions, electronic band structure, chemical composition, and the influence of synthetic routes, such as solution processing or mechanochemical methods, provides insights into the origin of the optical properties of the resulting WLE-LDHPs.^80–83^ The impact of compositional variations, particularly halide substitution (e.g., Cl, Br, I), on the emission properties is of great interest, as they can significantly alter the bandgap, exciton binding energy, emission wavelength, self-trapping depth, and trapping and de-trapping processes. However, challenges in obtaining an isostructural series of WLE-LDHPs led to limited studies of the halides’ role in these processes. In this work, we expand upon a previous study of (2,5-dmpz)­PbX_4_, (2,5-dmpz = 2,5-dimethylpiperazine), a series of iso-structural one-dimensional WLE-LDHPs consisting of Cl, Br, and I compounds.^83^ These compounds form as edge sharing octahedral dimers, which are further extended to 1D wires through corner sharing. The edge sharing dimers are associated with the formation of STE emission based on the enhanced broad emission of the (2,5-dmpz)­PbBr_4_ compound compared with (2,6-dmpz)­PbBr_4_, which is composed of mixed monomer and dimer wires. The photoluminescence quantum yield (PLQY) of the (2,5-dmpz)­PbBr_4_ compound is twice the value of (2,6-dmpz)­PbBr_4_.^50,64^

We began by synthesizing a series of pure halide samples—(2,5-dmpz)­PbCl_4_, (2,5-dmpz)­PbBr_4_, (2,5-dmpz)­PbI_4_. All of these compounds show broad STE emission (Figure S1). Interestingly, we noticed that their STE emission exhibits noncorrelative behavior with their onset energy. As the onset increases, the STE emission is shifted to lower energies, resulting in a very large Stokes shift (∼1.68 eV for the Cl sample). We noticed that (2,5-dmpz)­PbCl_4_ and (2,5-dmpz)­PbBr_4_ exhibited bright emission in comparison to the much weaker emission of (2,5-dmpz)­PbI_4_. Hence, we expanded our study toward the series of mixed halide compounds based on Br and Cl—(2,5-dmpz)­Pb­(Br_*x*_Cl_1–*x*_)_4_. We focus our research on the synthesis of these compounds utilizing different mechano-synthesis approaches. We examine the effect of the composition and the synthetic route on their chemical properties, optoelectronic characteristics, and stability.

## Materials and Methods

Hydrochloric acid (HCl, 37 wt %), hydrobromic acid (HBr, 48 wt %), ethanol, diethyl ether, PbBr2 (98%), PbCl2 (99.9%), and *N*,*N*-dimethylformamide (99%) were purchased from Sigma-Aldrich, Fisher, Acros, or Alfa Aesar and used without further purification. 2,5-dimethylpiperazine (2,5-dmpz 98%) was purchased from Alfa Aesar and converted to halide salts for the mechanochemical synthesis as described below.

### Preparation of (2,5-dmpz)­Br_2_ and (2,5-dmpz)­Cl_2_ Salts

One gram of 2,5-dmpz (8.76 mmol) crystals was dissolved in 3 mL of ethanol under vigorous stirring at room temperature. After the crystals fully dissolved, 1 mL of the corresponding concentrated acid (HBr, 8.84 mmol or HCl, 12 mmol) was added, and a precipitate formed. The resultant precipitate, in the form of white crystals, was washed with diethyl ether and dried for 3 h under vacuum and then overnight in the hood.

### Mechanochemical Manual Grinding Synthesis of (2,5-dmpz)­Pb­(Br_*x*_Cl_1‑*x*_)_4_ (*X* = 0 to *X* = 1 with 0.1 Increments)

To form 1 mmol of WLE-LDHP polycrystalline powder, appropriate molar ratios of PbBr_2_, PbCl_2_, (2,5-dmpz)­Br_2_, and (2,5-dmpz)­Cl_2_ along with 100 μL of DMF were manually ground together by using a mortar and pestle for 15 min. The formation of WLE-LDHPs was confirmed by broad emission under 365 nm of a commercial UV lamp.

### Mechanochemical Ball Milling Synthesis of (2,5-dmpz)­Pb­(Br_*x*_Cl_1–*x*_)_4_ (*X* = 0 to *X* = 1 with 0.1 Increments)

To form 1 mmol of WLE-LDHP polycrystalline powder, appropriate molar ratios of PbBr_2_, PbCl_2_, (2,5-dmpz)­Br_2_, and (2,5-dmpz)­Cl_2_ along with 100 μL of DMF were added to 2 mL tubes and mixed together with 1 mm ceramic balls in a commercial ball milling machine (CY Scientific Instruments). The samples were milled in three cycles, each cycle lasting 90 s to control the heating of the tube and sample during the process. The formation of WLE-LDHPs was confirmed by broad emission under a UV lamp.

### Absorption Measurements

Diffusive reflectance was measured using a Jasco V-760 UV–vis spectrophotometer with an integrating sphere, operating in the range of 250–1500 nm using BaSO_4_ as the reference of 100% reflectance. The absorption was obtained by converting reflectance to absorption according to the Kubelka–Munk equation: α_(λ)_/*S* = (1–*R*_(λ)_)^2^/(2*R*_(λ)_), where α is the absorption coefficient, *S* is the scattering coefficient, and *R* is the recorded reflectance.

### Photoluminescence Measurements

Photoluminescence spectra were recorded by using a Shimadzu RF-6000 spectrofluorometer. The excitation wavelength was set to 280 nm for all samples, and emission spectra were collected in the range of 320–900 nm using a 310 nm long-pass filter. The slit widths for excitation and emission were adjusted based on the emission intensity of each sample.

### Photoluminescence Quantum Yield Measurements (PLQY)

Photoluminescence spectra were measured by using an Edinburgh Instruments FS5 spectrometer equipped with an integrating sphere. The samples were excited at 280 nm, and the spectra were measured between 250 and 900 nm to include both the scattering signal from the lamp and the samples’ emission. The PLQY is calculated based on the ratio between the integrated emission (350–900 nm) and the integrated difference of the scattered photons (260–300 nm), representing the number of absorbed photons.

### Powder X-ray Diffraction Measurements (pXRD)

Powder X-ray diffraction patterns of the samples were collected using a Bruker AXS D8 Advance X-ray diffractometer equipped with a Cu Kα radiation source (λ = 1.5409 Å). Measurements were conducted over the 2θ range of 2–60° utilized to identify crystalline phases, calculate lattice parameters, and evaluate the structural purity of the samples.

### Thermogravimetric Analysis (TGA)

TGA spectra were measured using a TA Instruments Q50 thermogravimetric analyzer. The samples were heated from room temperature to 700 °C at the rate of 5 °C/min under N_2_ atmosphere.

## Results and Discussion

In order to thoroughly investigate the effect of halides, a precise control over the resulting compounds’ halide composition is essential. To achieve such control and consistency, we choose to utilize mechanochemical synthesis instead of the commonly used solution-based crystallization. The main advantage of mechanochemical synthesis is the avoidance of issues related to different solubilities and reaction kinetics of the precursors and the resulting compounds. For these reasons, (2,5-dmpz)­Pb­(Br_*x*_Cl_1–*x*_)_4_ (*x* = 0 to 1 in increments of 0.1) samples were prepared employing two mechanochemical routes: manual grinding with a mortar and pestle and mechanical grinding with a commercial ball milling machine. Both grinding processes involved combining (2,5-dmpz)­X_2_ and PbX_2_ salts (X—Cl/Br), with a few drops of DMF to facilitate the formation of the desired WLE-LDHPs in the form of white, polycrystalline powders. It is important to note that the DMF serves as a milling agent and does not fully dissolve the precursors. The structure of the as-synthesized crystalline powders was confirmed by powder X-ray diffraction (pXRD) measurements. These compounds adopt a one-dimensional (1D) structure in the form of double edge-sharing octahedra that extends through corner-sharing connections,^83^ with the edge-sharing octahedral dimers and corner-sharing connectivity as the key structural motifs.

Based on the reported structures of pure halides, the characteristic pXRD peaks are expected to appear at 2θ ∼ 8° (dimer width) and 2θ ∼ 12.5° (octahedral size). The resulting pXRD patterns of the two series, formed by manual grinding and ball milling, are displayed in Figure 1A,B. Both match the reported patterns for the pure halide compounds and show the expected characteristic diffraction peaks (Figure S2). Gradual shifts in the diffraction peaks are observed due to variations in the lattice parameters of the unit cell based on the halide composition. As expected, the unit cell parameters correspond with the halide radii, resulting in a shift toward higher angles (smaller unit cell) for the samples with larger Cl content. Only the relative intensities and peak widths of the diffraction patterns show minor differences between samples synthesized via different routes, which can be attributed to variations in their particle size distribution. When the two synthesis methods are compared, the resulting diffraction patterns of the mixed halide (2,5-dmpz)­Pb­(Br_*x*_Cl_1–*x*_)_4_ compositions prepared via manual grinding exhibit stronger peaks compared to those synthesized using ball milling, which produces sharper, narrower peaks but with lower intensity. In manual grinding, the force applied on the sample is limited by the user. Moreover, the mechanical force is not applied consistently. Both of these effects tend to result in a less efficient size reduction process, coarser grinding, and, hence, larger particle size. The larger particles generally contain a greater number of crystallographic planes that can contribute to constructive interference during diffraction, resulting in increased peak intensity. However, upon closer examination of the diffraction patterns from both synthesis methods, it is evident that the diffraction peaks of the samples produced by ball milling are better resolved. For example, in the region of 2θ = 22.5°, the ball milling samples exhibit only two distinct peaks, whereas those prepared by manual grinding display a number of additional small peaks overlapping each other in this region (see Figure 1A,B).

**Figure 1:**
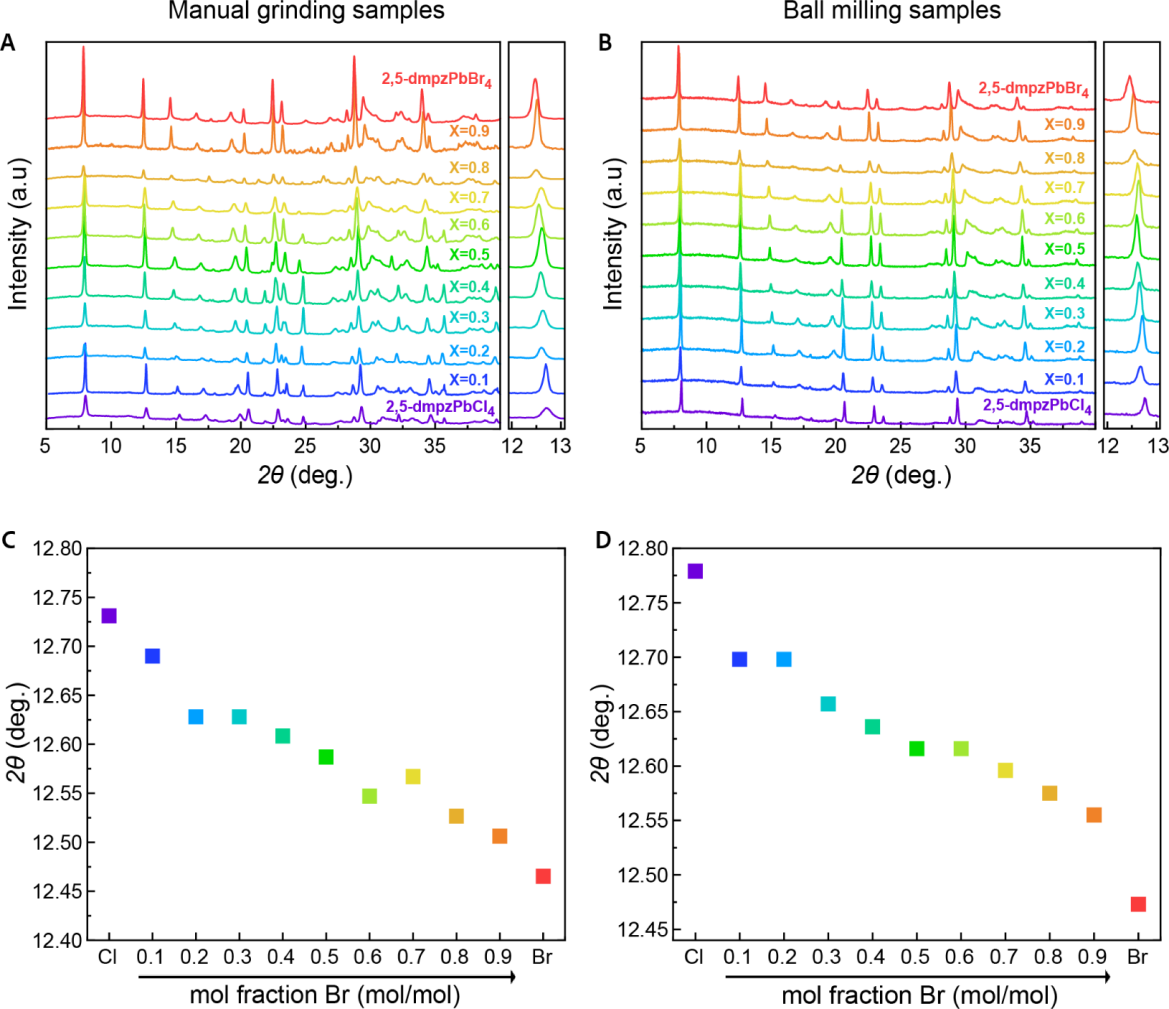
Structural characterization of manually ground samples (A, C) and ball milling samples (B, D). Stacked pXRD patterns (A, B) of the alloyed samples prepared by manual grinding and ball milling, respectively. Variation of the 2θ angle of a characteristic diffraction peak as a function of halide composition for the manually ground (C) and ball milled (D) samples.

These observations indicate that the ball milling process promotes the formation of more homogeneous, ordered, and crystalline powders, compared to manual grinding. This is likely due to the enhanced homogeneity and efficient particle size distribution achieved during motorized mechanical ball milling. The forces operate on the sample, and the increased temperature during ball milling facilitates more uniform mixing of the elements, improving the crystallinity of the resulting materials. In Figure 1C,D, we follow a typical diffraction peak of the octahedral size and plot its angle as a function of the bromide molar fraction (*x*), illustrating the structural trend described above. These plots show the expected structural trend; however, it seems like there are some deviations from the expected linear dependency, suggesting that some nonlinearity may also exist in the optical characteristics.

The optical properties of the two alloyed series (manually ground and ball-milled) were evaluated by using absorption and photoluminescence (PL) spectroscopy of the polycrystalline powders. Absorption measurements of these solid samples were analyzed in order to determine the absorption onset and exciton energy of each compound. The absorption onset values were calculated by extrapolation of the linear rise of the absorption peaks (see Tables S1–S6). As shown in Figure 2A,B, all samples exhibit clear band edges, and the spectral shapes above the absorption onset are typical for 1D electronic structures. For both (2,5-dmpz)­Pb­(Br_*x*_Cl_1–*x*_)_4_ series, a systematic shift in the absorption onset toward lower energies is observed with increasing Br content, aligning with previous reports on low-dimensional mixed halide perovskites.^84,85^

**Figure 2:**
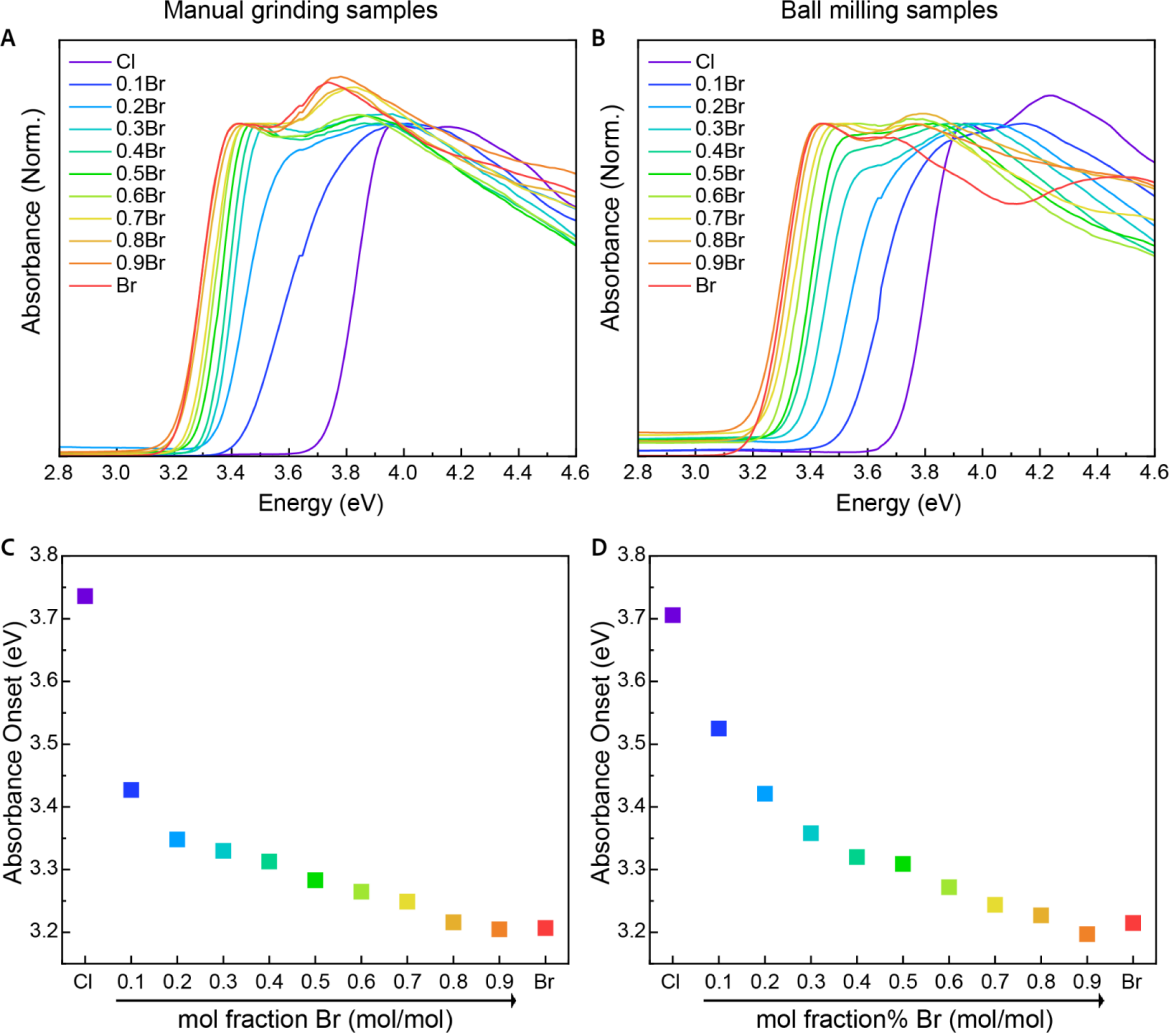
Absorption measurements of manually ground samples (A, C) and ball milling samples (B, D). (A, B) The resulted absorption spectra of the manually grinded and ball milling samples, respectively. (C, D) Extracted absorption onset values of the manually grinded and ball milling samples, respectively, plotted as a function of halide composition.

The variations in the onset values are plotted in Figure 2C,D as a function of composition. In both synthetic routes, fine-tuning of the onset is achieved, with the values decreasing from 3.7 to 3.2 eV as the Br content increases. Notably, a sharp decrease in the absorption onset value is observed upon transitioning from the pure chloride phase to the mixed phase (2,5-dmpz)­Pb­(Br_0.1_Cl_0.9_)_4_. This rapid change is more pronounced in the samples prepared by manual grinding, supporting the assumption that the mixing is less efficient and the effective molar ratios in these compounds slightly differ from the expected ones. In the samples prepared by ball milling, the following two transitions, Br_0.1_Cl_0.9_ → Br_0.2_Cl_0.8_ and Br_0.2_Cl_0.8_ → Br_0.3_Cl_0.7_, also exhibit fairly large changes, with the onset shifts of 0.1 and 0.05 eV, respectively, compared to the other transitions, that show shifts of approximately 0.02 eV. This observation suggests that chloride may be more dominant than bromide in dictating the optical bandgap. Plotting the absorption onsets as a function of Br content shows a bowing instead of a linear trend, emphasizing a nonlinear relationship between composition and the magnitude of the onset.^86,87^

PL measurements were conducted to investigate the relaxation from the excited state and, specifically, the STE characteristics of the mixed halide series. As shown in Figure 3, for both ball milling and manual grinding synthesis, the single halide compounds exhibit dual emission from both the FE and STE energy states. The narrow emission peaks at 3.29 eV for ball milling (3.26 eV in manual grinding synthesis) and 3.724 eV (both methods) for the pure bromide and chloride compounds, respectively, are attributed to FE emission. The FE PL peaks closely match the absorption onsets determined from the absorption spectra (see Tables S3, S6). Consistent with the absorption behavior, the FE peak of the pure bromide sample is red-shifted relative to that of the pure chloride sample. On the other hand, the broad emission peaks reveal an anticorrelation between the STE energy and the onset. The STE peaks are located at 2.465 eV for ball milling (2.445 eV in manual grinding synthesis) and 2.37 eV (both methods) for the pure bromide and chloride, respectively, indicating that the STE emission of the pure bromide compound with the smaller onset is blue-shifted relative to the pure chloride sample with the higher onset.

**Figure 3:**
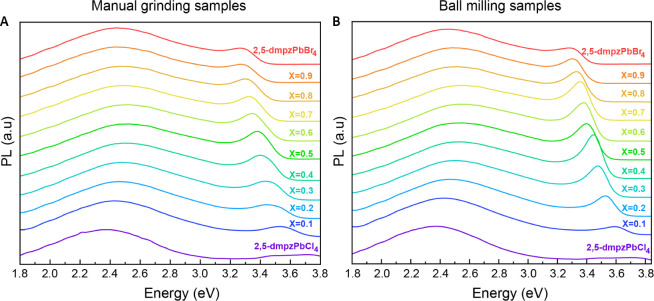
PL measurements of manually ground samples (A) and ball milling samples (B). All samples exhibit dual emission from FE and STE.

To settle this apparent contradiction, we discuss the origin of STE and its correlation to the local properties of the crystal lattice rather than to the electronic band structure dictated by long-range order. In LDHPs, as dimensionality decreases, the number of degrees of freedom of the octahedra increases, resulting in a greater likelihood of structural distortions, primarily through octahedral tilting that alters bonding angles. These crystal lattice distortions reduce orbital overlap and increase phonon dispersion, thereby limiting charge carrier mobility and enhancing exciton–lattice coupling. The coupling induces transient changes in the local potential, resulting in the formation of a potential well, characterized by lower energy levels than those of FE, enabling exciton trapping and confinement within the potential well. Excitons with sufficient energy can overcome the potential barrier separating FE and STE and a transition from the excited state to STE states. Thus, STE emission is characterized by energy loss, observed as a red shift of the STE emission relative to the FE emission corresponding to the band edge. Hence, the observed anticorrelation between onset energy and the STE spectrum is associated with the different origins for each, e.g., Cl will lead to wider onset energy and more rigid structure, leading to larger energy loss in the trapping process. Indeed, the measured energy loss is smaller in softer materials. The energy difference between the FE and STE peaks in the softer bromide compound is 0.825 eV for ball milling and 0.815 eV for manual grinding compared with 1.35 eV for the more rigid chloride compound.

The mixed halide compounds exhibit the same spectral shape with both FE and STE emission. The FE emission in these compounds monotonically decreases between the typical values for pure chloride and pure bromide as the Br content increases, following the expected behavior based on the onset energies. However, the STE emission shows a surprising deviation from the anticipated trend. Initially, increasing the bromide content leads to a blue shift of the STE emission energy, as expected. Yet, the blue shift is largest for the ball milling sample with *x* = 0.6, exhibiting STE emission centered at an energy of 2.55 eV (*x* = 0.5, 2.5 eV for manual grinding). For samples with higher Br content, this trend reverses, and the emission begins to red shift. This suggests that the combination of two halides at the X site produces a softer structure compared with each of the pure halides, resulting in a smaller STE energy shift and an overall blue shift of the STE emission. In addition, the shape of the plotted curves reveals a nonlinear correlation between composition and STE emission in both synthetic routes. When comparing the synthetic methods, the compounds prepared via mechanical ball milling show higher STE emission energies and a more symmetric optical trend compared to those prepared by manual grinding (see Figures 4 and S6). This further emphasizes the significant influence of the preparation method and conditions on the optical properties of these materials.

**Figure 4:**
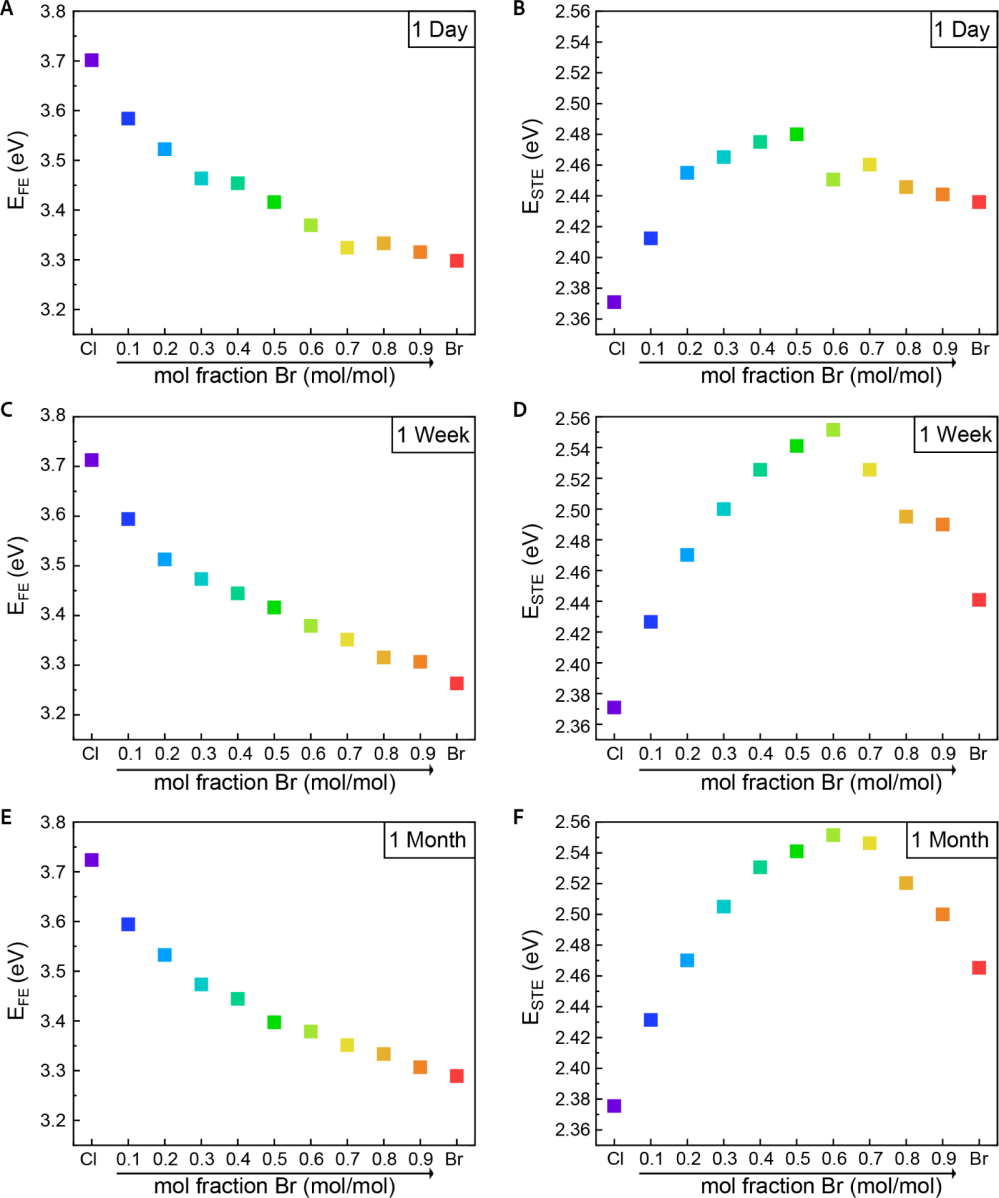
PL trends of the ball milling samples. (A, C, E) The FE emission peaks after 1 day, 1 week, and 1 month, respectively. (B, D, F) The development of the STE emission peaks after 1 day, 1 week, and 1 month, respectively.

Moreover, the STE emission exhibits another peculiar behavior; the shape and energy of the STE emission changed and evolved with time after synthesis. To assess this observation, we measured the PL, the absorption, and the pXRD of the ball milling samples on the same day of preparation, after 1 week and after 1 month. While an evolution of the emission over time is clearly observed for the STE, the FE emission showed no significant change over time, both in the spectral shape and in the energies (Figure 4). However, the overall scale of the emission remains consistent, with energy shifts on the order of few meV. This suggests that the optical bandgap, corresponding to the FE emission, remains stable over time.

In contrast, the STE emission undergoes substantial changes in both the spectral shape and peak energy over time. As shown in Figure 4B, after 1 day, the STE emission exhibits a nonlinear blue shift, peaking at an energy of 2.48 eV for the sample with *x* = 0.5, followed by a linear red shift for samples with higher bromide content. After 1 week (Figure 4D), the span of the STE emission energies extends, driven by larger energy differences between the mixed halide samples, while the pure halide samples remain unchanged. This results in a blue shift, peaking at 2.55 eV for the sample with *x* = 0.6, and a pronounced linear red shift for samples with higher bromide content. By the end of one month (Figure 4F), the spectral shape appears to stabilize, with both the blue and red shifts exhibiting similar compositional dependencies, with a degree of bowing. Notably, after this time, the STE energies remain approximately constant, and the PL spectra show no further variation, supporting the conclusion that the STE emission has reached a steady state. The evolution of the STE emission, in contrast to the steady state of the FE emission, indicates that any modifications occurring in these alloys over time are associated with local structure and not with the long-range ordering.

To further study the time evolution, complementary absorption measurements were conducted. The results exhibit similar behavior to the FE PL, mostly minor variations are seen, and no significant changes in the composition or in the onset energies are observed. As mentioned, the overall spectral trend remains the same over time without a change in the bowing effect. For the manually grinded samples, the STE and FE emissions exhibited similar evolutionary trends to those observed in ball milling samples (see Figure S6); however, the processes appeared to progress at a significantly slower rate. The FE emission exhibited negligible energy shifts over time, while the STE emission exhibited a more linear blue and red shift, with a pronounced curvature developing only after one month. In contrast, the ball milling samples reached comparable optical trends much earlier, with stabilization occurring within 1 week. These differences highlight the significant influence of the preparation method on the STE evolution kinetics.

In addition, we compared the pXRD spectra for the pure halides and three mixed halide samples (*x* = 0.1, 0.5, 0.9), taken after 1 day, 1 week, and 1 month. These measurements show consistent structural peaks at the same positions for all time periods (Figures S7–S12), further supporting the hypothesis that the STE evolution is related to local lattice variations and not modifications of the crystal structure. Mixed halide perovskites with high Cl content often face stability challenges due to their strong tendency for phase segregation. However, in our series of materials, compounds with high Cl content demonstrated notable structural stability, showing no evidence of phase segregation. While the general trend is clear, we did observe minor differences in the pXRD patterns between the different compositions and the synthetic methods as highlighted below.

For the compound (2,5-dmpz)­Pb­(Br_0.1_Cl_0.9_)_4_, with the highest Cl content, the sample prepared by manual grinding showed no observable changes in the pXRD patterns over time (see Figure S12A). In contrast, the sample prepared by ball milling exhibited an increase in diffraction peak intensity over time, while peak widths remained unchanged, suggesting enhanced crystallinity or improved ordering without significant structural broadening or changes in particle size (Figure S8). For the (2,5-dmpz)­Pb­(Br_0.5_Cl_0.5_)_4_ sample, the trend is reversed; manually grinded samples exhibited an increase in peak intensity over time (see Figure S12B), while ball milling samples showed a decrease (see Figure S9). The trends observed for (2,5-dmpz)­Pb­(Br_0.9_Cl_0.1_)_4_ compounds also differ between the two synthetic routes. In both cases, there is a decrease in peak intensity over time. This further emphasizes the impact of the preparation method on the structural stability of the material. Despite these differences, the magnitude of the changes is minor, making it reasonable to conclude that the structure remains the same over time in all samples. In addition, the fact that among the various properties examined—crystal structure, absorption, FE emission, and STE emission—only the STE emission evolves over time, indicating that certain characteristics are tied to the overall macrostructure and are less affected by time, whereas others are related to the microstructure at the atomic level and are more prone to time-dependent changes. The onset and the FE emission are related to the electronic structure, which is dictated by the long distance symmetry of the crystal structure. Consequently, they do not change over time. On the other hand, the broad emission associated with STE is a local phenomenon and is related to the microscopic atomic arrangement of the compound, which seems to evolve post-synthesis.

The ball milling system demonstrated a more efficient mixing process and quickly reached a steady state. For these reasons, this system was chosen for further exploration. To investigate atomic-level modifications over time, we utilized previous pXRD measurements combined with Williamson–Hall (W–H) analysis. This method provides a way to separate the effects of crystallite size and lattice microstrain on the broadening of X-ray diffraction peaks based on their distinct angular dependencies according to the following equation:^88,89^

βcosθ=DKλ+4εsinθ

where β is the full width at half-maximum (fwhm) of the diffraction peak in radians, θ is the Bragg angle, *K* is the shape factor (typically ∼0.9), λ is the X-ray wavelength, *D* is the crystal size, and ε represents the microstrain. By plotting βcos θ against 4sin θ, we obtain the crystallite size from the y-intercept, while the slope gives the microstrain. The calculated values of microstrain and crystal size are summarized in Table 1, with the observed trends illustrated in Figure 5. Notably, the single halide compounds serve as a reference for the magnitude of microstrain evolution over time of the mixed halide samples. For the chloride-based compound, the microstrain changes from a magnitude of 171 after 1 week to 169 after 1 month, remaining fairly constant over time. Similarly, the bromide-based compound shows microstrain values of 168 after 1 week and 167 after 1 month, thus also not exhibiting evolution over time. Hence, the pure halide compounds exhibit the lowest microstrain and remain relatively constant over time. The mixed compounds with molar fractions *x* = 0.1 and *x* = 0.9 exhibit slightly elevated values compared to the pure phase ones. The (2,5-dmpz)­Pb­(Br_0.9_Cl_0.1_)_4_ sample shows no significant change over time with microstrain of 192 to 193 and 189 after 1 day, 1 week, and 1 month, respectively, and these values are higher than those of the pure bromide sample. The (2,5-dmpz)­Pb­(Br_0.1_Cl_0.9_)_4_ sample exhibits a decrease in the microstrain and fairly significant change between the time intervals of 1 day and 1 week, changing from 187 to 171. However, after 1 week, it appears to reach equilibrium and remains at a value of 171, similar to the pure chloride compound. Among the samples, the highest magnitude of microstrain is observed for (2,5-dmpz)­Pb­(Br_0.5_Cl_0.5_)_4_, significantly higher than that of the other mixed halides and the pure samples. It exhibits a unique behavior of nonmonotonic evolution over time with an initial increase from 235 to 248 after 1 week, followed by a decrease to a value of 217 after one month.

**Figure 5:**
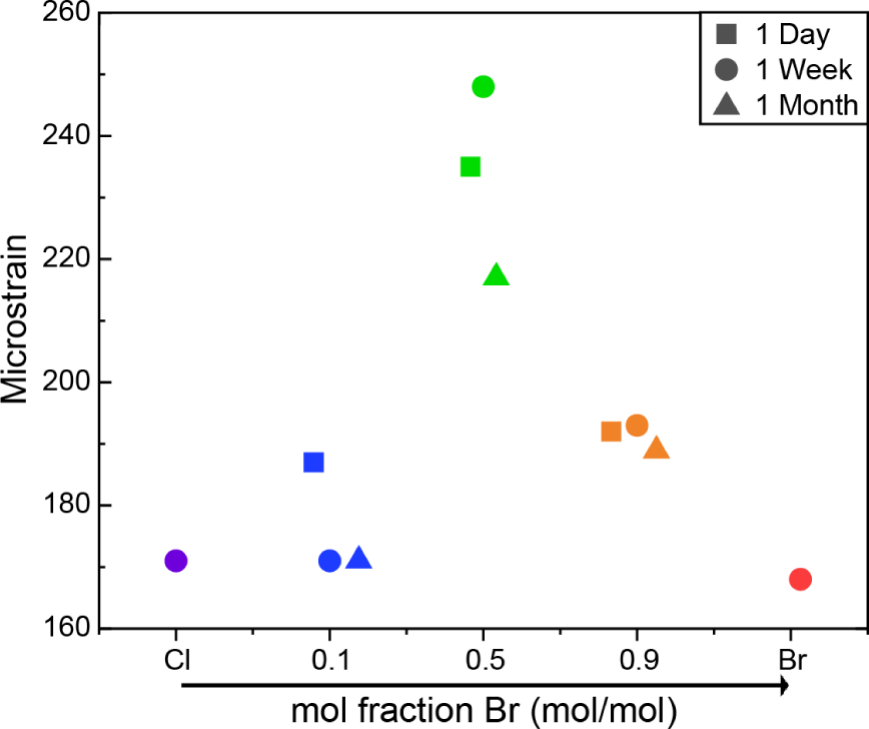
Microstrain measurements of (2,5-dmpz)­PbCl_4_, (2,5-dmpz)­Pb­(Br_0.1_Cl_0.9_)_4_, (2,5-dmpz)­Pb­(Br_0.5_Cl_0.5_)_4_, (2,5-dmpz)­Pb­(Br_0.9_Cl_0.1_)_4_, and (2,5-dmpz)­PbBr_4_ compounds prepared by ball milling.

**Table 1:** Calculated Microstrain Values and Crystal Size of *x* = 0, 0.1, 0.5, 0.9, 1

		Cl	*x* = 0.1	*x* = 0.5	*x* = 0.9	Br
**1 day**	microstrain (ε)		187	235	192	
	crystal size (nm)		188	187	166	
**1 week**	microstrain (ε)	171	171	248	193	168
	crystal size (nm)	160	177	263	173	82
**1 month**	microstrain (ε)	169	171	217	189	167
	crystal size (nm)	70	173	204	180	97

Consequently, while the overall crystal symmetry appears to remain unchanged, the local structural modifications occurring over time likely contribute to the observed changes in the STE emission properties. This analysis supports our hypothesis that halide mixing occurs in a manner that mirrors the properties of the pure compounds. Starting with the pure chloride compound, the introduction of bromide content progressively shifts the material’s properties toward those of the pure bromide compound. Similarly, for the pure bromide sample, the addition of chloride content leads the material to resemble that of the pure chloride compound. The behavior observed in the Br_0.5_Cl_0.5_ ratio, however, deviates from the general trends and is not fully understood; hence, an additional analysis is required to draw conclusive insights regarding the atomic rearrangement in mixed halide LDHPs.

To further analyze the samples’ suitability for future optoelectronic applications, we have studied their stability at elevated temperatures by thermogravimetric analysis (TGA). Each sample was heated from room temperature up to 700 °C under inert gas atmosphere (N_2_) while monitoring the weight to identify decomposition and evaporation of the sample. The TGA results show that each sample undergoes two mass loss events (Figure S13). The first mass loss event at 300–350 °C is associated with decomposition of the 1D structure followed by evaporation of the (2,5-dmpz) molecules and part of the halide atoms. This decomposition is leading to the formation of Pb-halide salts, which are stable up to 600–650 °C; above this temperature, a second mass loss event occurs upon the complete evaporation of the Pb-halide. To further study this process, the portion of mass loss for the first and second events was calculated for each sample. We then modeled two possible decomposition paths based on halide mixing—either Cl is leaving before Br (due to higher volatility) or the halides are coevaporating based on the samples’ composition (Figure S14). Based on the TGA results and our model, we found that during the first decomposition event, Cl is initially consumed, leaving behind a Br-rich PbX_2_ phase. Hence, for compositions up to *x* = 0.5, the first mass loss is of 2,5-dmpz + Cl_2_, while the second mass loss is of Pb­(Br_2*x*_Cl_1–2*x*_)_4_. For *x* > 0.5, the first mass loss is of 2,5-dmpz + all Cl_2_ + some of Br_2_; the second mass loss event is of pure PbBr_2_. To study how alloying is affecting the decomposition temperature, we extracted the temperatures of the first and second events (by plotting the derivative of the TGA plot—Figure S15). These temperatures agree with our former analysis of the decomposition process—for *x* < 0.5, the first event (associated with the phase of pure composition) occurs at constant temperature, while the second event (associated with phases of mixed composition) becomes lower as the Br content is higher, in agreement with the higher melting and boiling temperatures of PbCl_2_ comparing to PbBr_2_. For compositions of *x* > 0.5, the temperature of the first event (associated with mixed Cl/Br composition) is increased as the Br content increases, while the temperature of the second event (associated with pure PbBr_2_) remains constant (Figure S16). This analysis highlights the excellent stability of these compounds, maintaining their structure up to a temperature of 300 °C.

Lastly, we assessed the STE emission intensity of the pure and mixed compound by PLQY measurements. For these measurements, the PL of each sample is measured inside an integrating sphere upon 280 nm excitation (see Figure S17). The integrated emitted photons are compared with the absorbed photons to calculate the PLQY value. The PLQY values of the ball milling samples vary between 11% and 35%, peaking for the sample with *x* = 0.3, as presented in Figure 6. Comparing to the PLQY of additional 1D lead-halide broad emitters (see Table S7), the PLQY of these samples is among the highest. Interestingly, the PLQY for ball milling (2,5-dmpz)­PbBr_4_ is lower than the reported value for solution-based (2,5-dmpz)­PbBr_4_.^50^ This phenomenon was also observed for other samples, showing higher PLQY for large crystals comparing to microcrystals such as the ones produced through ball milling.^55^ Moreover, it may suggest that by tuning the synthetic procedures of the mixed halide samples, even higher PLQY values are expected.

**Figure 6:**
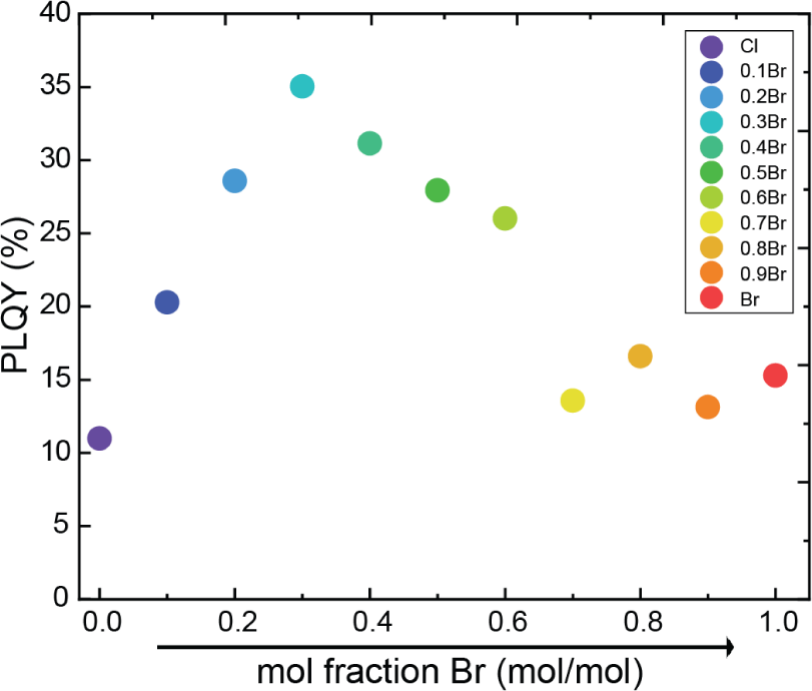
PLQY values of (2,5-dmpz)­Pb­(Br_*x*_Cl_1–*x*_)_4_ samples synthesized by ball milling. Measured upon excitation at 280 nm.

## Conclusions

This study provides a detailed investigation into the effects of halide composition and preparation methods on the optical and structural properties in mixed halide perovskites. We have demonstrated that although the STE emission is dictated by specific crystal structures, its fine-tuning is primarily governed by the atomistic nature of the material. Moreover, the STE emission energy is defined by the atomistic properties and not by the compound’s electronic structure. Hence, the STE emission properties are influenced by the chosen synthetic path and the preparation method of the material. We observed significant differences between the two synthetic methods—mechanical ball milling and manual grinding. The ball milling method proved to be advantageous, resulting in higher crystallinity of the powders, symmetric trends in the optical characteristics, and faster evolution of the STE properties over time. This indicates that ball milling facilitates an efficient mixing process and better integration of the halides at the atomic level. Temporal evolution of the STE emission demonstrated nonlinear changes, with notable differences observed between one-day, one-week, and one-month intervals. This time-dependent behavior further supports the hypothesis that atomic arrangements drive changes in the STE emission rather than macroscopic structural changes. The analysis of microstrain across various compositions of mixed halide perovskites reveals that the microstrain is observed to be the highest for *x* = 0.5, where it is significantly higher than for *x* = 0, *x* = 0.1, *x* = 0.9, and *x* = 1, which remain relatively constant over time. These findings support our hypothesis that the mixing of halides is gradually evolving post-synthesis, with the material shifting toward the properties of the dominant halide in the mixture. To assess these samples’ suitability for future optoelectronic devices, we have measured their thermal stability and emission efficiency. These samples are stable up to a temperature of 300 °C, which is sufficient even for demanding light-emission applications. Finally, these samples showed strong emission with a PLQY of up to 35%, among the highest measured for 1D samples with broad emission. All of these indicate the potential of utilizing these compounds for efficient light emission applications.

## Supplementary Material



## Notes

Additional optical characterization of pure halide compounds and the mechanically ground samples; data tables of extracted absorption and PL energies for all samples, and comparison of the optical properties for various 1D lead halide broad emitters; figures of the W–H microstrain analysis, pXRD spectra of the mechanically ground samples, TGA spectra and analysis, and PLQY spectra (PDF)
