# Supplementary material for: Tuning Self-Trapped Exciton Emission in 1D White-Light Emitting Perovskites Through Halide Composition and Synthesis Route
Source: ACS Omega. 2025 Jun 9;10(24):25708–19. doi: 10.1021/acsomega.5c01452 (PMC12199075; doi:10.1021/acsomega.5c01452)
Supplement: Supplementary file 1 [file ao5c01452_si_001.pdf]

Supplementary Information

Tuning Self-Trapped Exciton Emission in 1D White-Light Emitting Perovskites Through Halide Composition and Synthesis Route

Bar Bader[1], Elisheva Michman[1], Ioannis N. Gkikas[2], Ioannis Spanopoulos[2], Ido Hadar[1]*

[1]Institute of Chemistry, The Center for Nanoscience and Nanotechnology, and Casali Center for Applied Chemistry, The Hebrew University of Jerusalem, Jerusalem, Israel, [2]Department of Chemistry, University of South Florida, Tampa, FL, USA

List of Content

Figure S1 – Absorption and PL spectra of pure (2,5-dmpz)PbX_4 samples (X = Cl, Br, I)

Figure S2 – pXRD measurements of pure (2,5-dmpz)PbX_4 samples (X = Cl, Br) synthesized by ball milling and comparison to reported structures

Figure S3 – Absorption spectra - manually grinded samples

Figure S4 – Absorption trends - manually grinded samples

Figure S5 – PL spectra - manually grinded samples

Figure S6 – PL time evolution- manually grinded samples

Table S1 – Summary of Abs and PL extracted/calculated values of the manually grinded samples after one day

Table S2 – Summary of Abs and PL extracted/calculated values of the manually grinded samples after one week

Table S3 – Summary of Abs and PL extracted/calculated values of the manually grinded samples after one month

Table S4 – Summary of Abs and PL extracted/calculated values of the ball milling samples after one day

Table S5 – Summary of Abs and PL extracted/calculated values of the ball milling samples after one week

Table S6 – Summary of Abs and PL extracted/calculated values of the ball milling samples after one month

Figure S7 – pXRD spectra and W-H linear fittings of pure (2,5-dmpz)$PbCl_4$ compound prepared by ball milling

Figure S8 – pXRD spectra and W-H linear fittings of pure (2,5-dmpz)$Pb(Br_{0.1}Cl_{0.9})_4$ compound prepared by ball milling

Figure S9 – pXRD spectra and W-H linear fittings of pure (2,5-dmpz)$Pb(Br_{0.5}Cl_{0.5})_4$ compound prepared by ball milling

Figure S10 – pXRD spectra and W-H linear fittings of pure (2,5-dmpz)$Pb(Br_{0.9}Cl_{0.1})_4$ compound prepared by ball milling

Figure S11 – pXRD spectra and W-H linear fittings of pure (2,5-dmpz)$PbBr_4$ compound prepared by ball milling

Figure S12 – pXRD spectra of the manually grinded samples after one day, one week, and one month

Figure S13 – TGA measurements of (2,5-dmpz)$Pb(Br_xCl_{1-x})_4$ compounds prepared by ball milling

Figure S14 – Mass loss of (2,5-dmpz)$Pb(Br_xCl_{1-x})_4$ compounds prepared by ball milling, measured and modeled

Figure S15 – Normalized TGA and TGA derivative of (2,5-dmpz)$Pb(Br_xCl_{1-x})_4$ compounds prepared by ball milling, and extracted decomposition temperatures

Figure S16 – Mass loss temperatures for (2,5-dmpz)$Pb(Br_xCl_{1-x})_4$ compounds prepared by ball milling

Figure S17 – PLQY measurements of (2,5-dmpz)Pb(Br_xCl_{1-x})$_4$ compounds prepared by ball milling

Table S7 – PLQY and optical properties of broad emitters with 1D structure

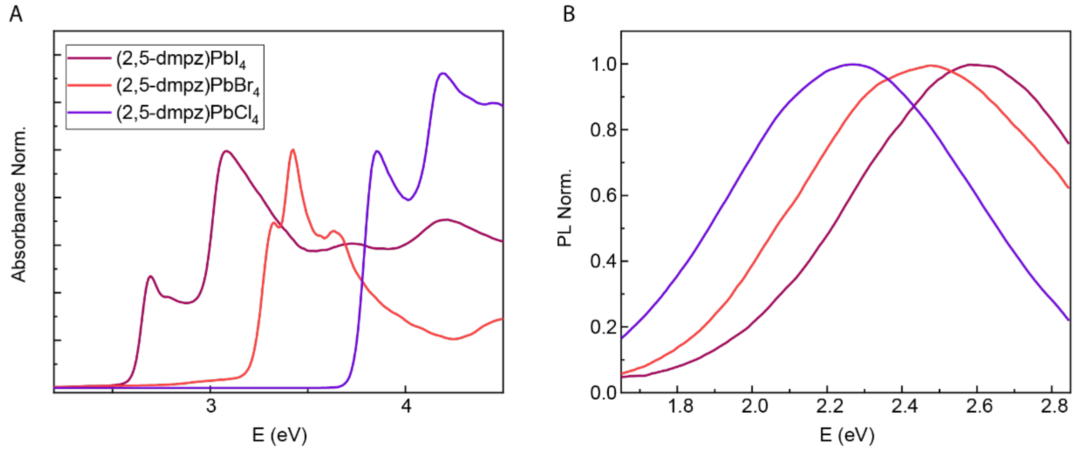

Figure S1. Absorption (A), and PL (B) spectra of pure (2,5-dmpz)PbX_4 samples (X = Cl, Br, I).

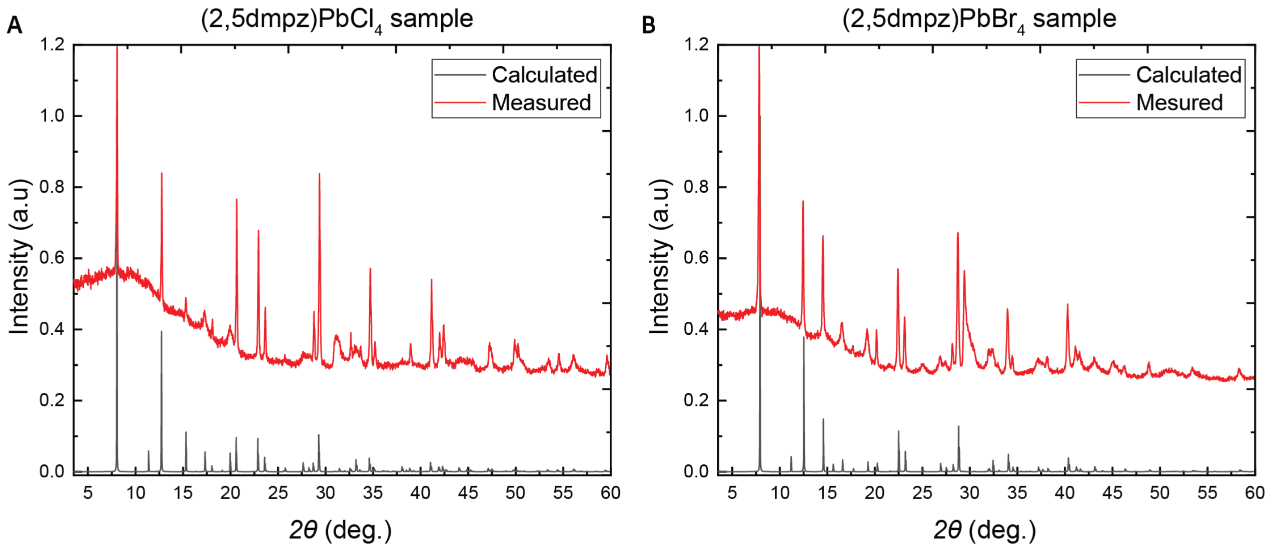

Figure S2. pXRD measurements of pure (2,5-dmpz)PbX_4 samples (X = Cl, Br) synthesized by ball milling and comparison to calculated pXRD based on reported structures.

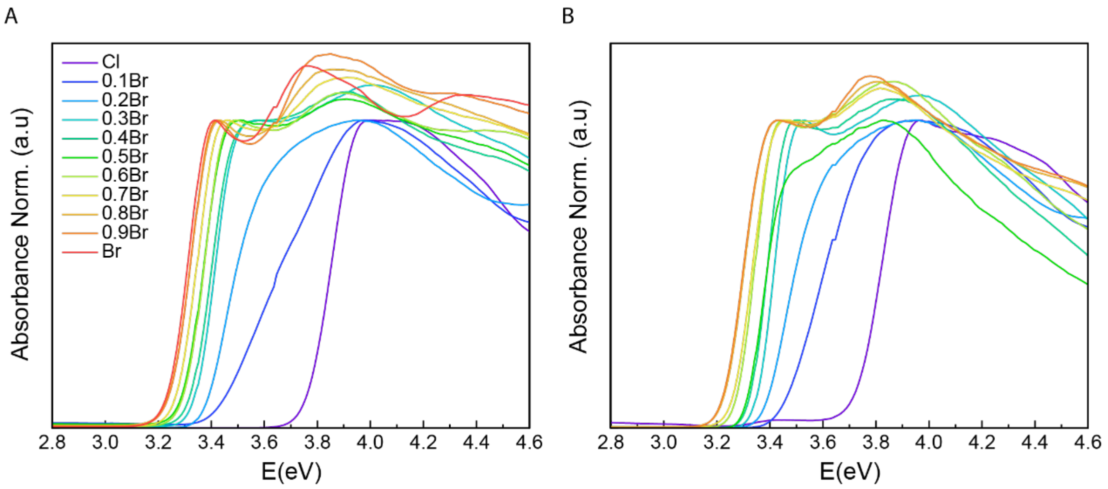

Figure S3. Absorption spectra of the manually grinded samples after one day (A) and after one week (B).

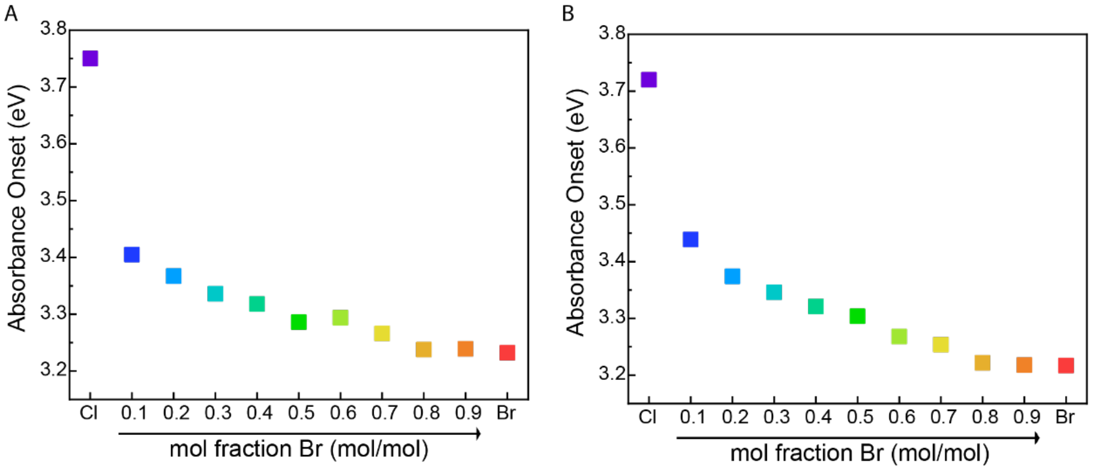

Figure S4. Extracted absorption onsets values of the manually grinded samples as a function of halide composition, after one day (A) and after one week (B).

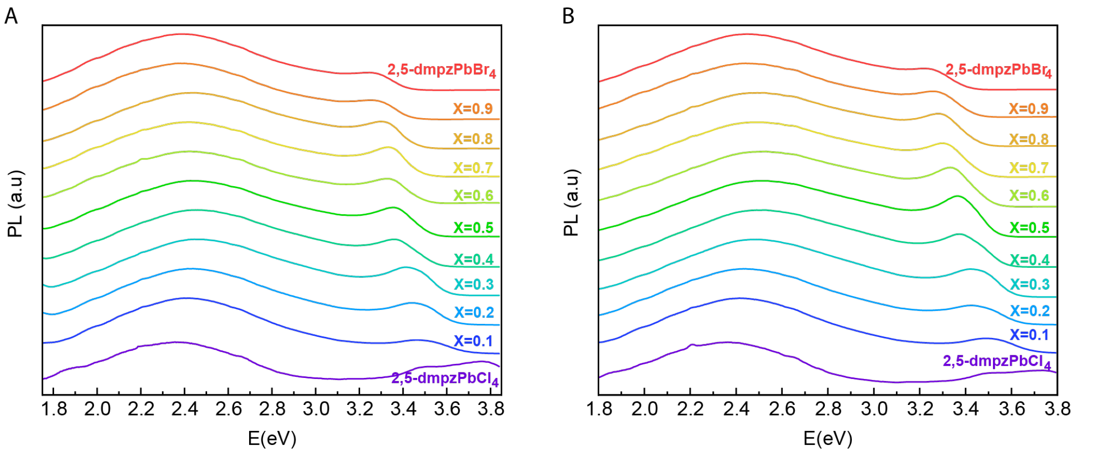

Figure S5. PL spectra of samples prepared by manual grinding after one day (A) and after one week (B).

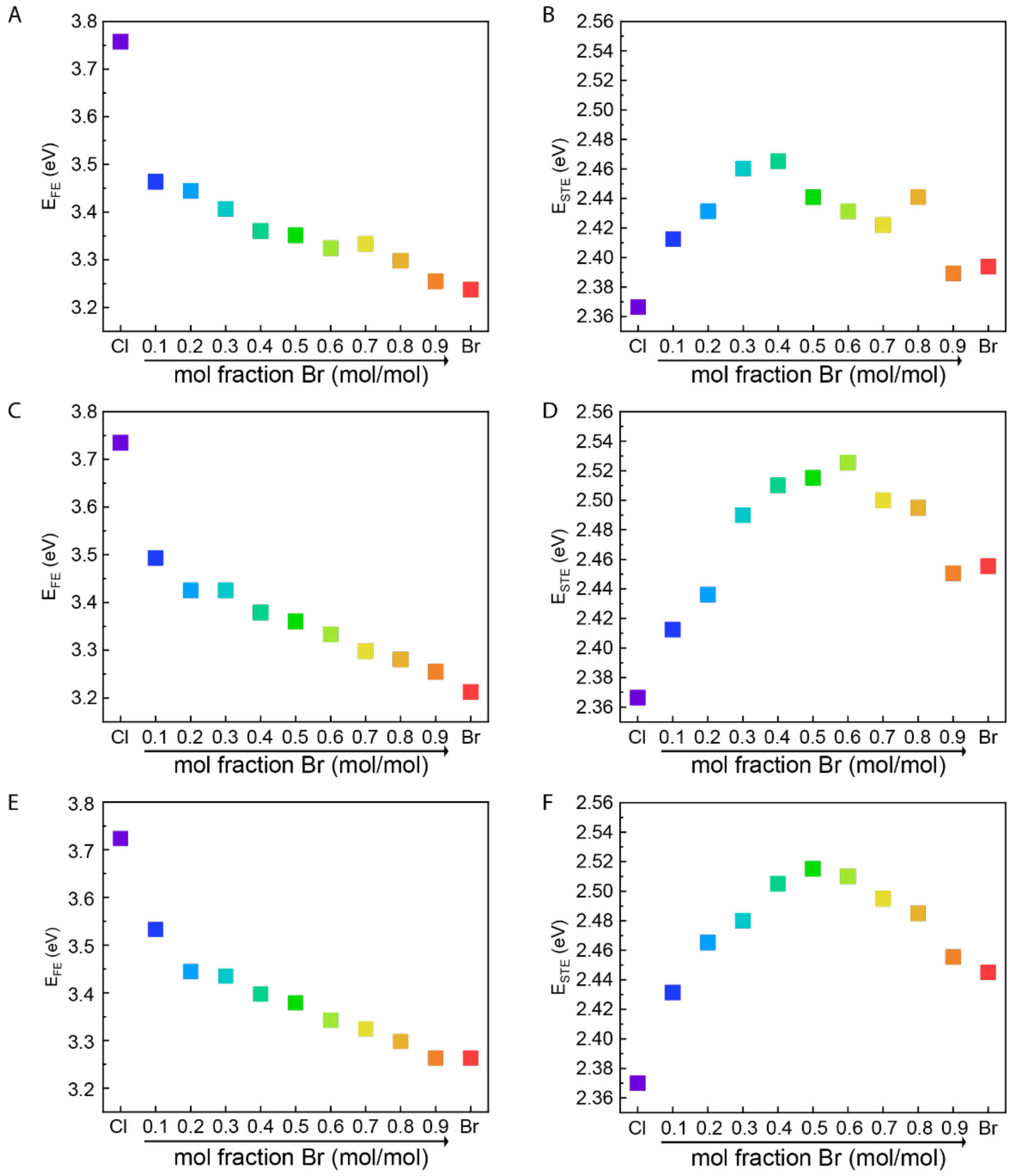

Figure S6. PL trends of the manually grinded samples. A, C, E, describe the FE emission peaks after one day, one week, and one month respectively. B, D, F describe the STE emission peaks after one day, one week, and one month respectively.

As shown in **Figure S6,** samples prepared by manual grinding exhibit a similar type of evolution in the STE emission as the samples prepared by ball milling. The STE spectra showed significant changes over time compared to the FE emission, however, in the manually grinded samples, both FE and STE emissions continued to evolve even after one month. Initially, the FE emission displayed a mostly linear decrease in energy from $x = 0.1$ to $x = 1.0$, with the pure chloride compound deviating from this trend by exhibiting a significantly higher energy. After one week, compounds with higher chloride content showed slight shifts to higher energies, while those with higher bromide content shifted to lower energies, narrowing the gap between the pure chloride compound and the other samples. After a month, the energy of the $x = 0.1$ compound continued to shift to higher energies, and the spectral shape began to display a bowing effect. However, compounds with higher chloride content did not appear to reach a steady state, as energy differences in this region remained relatively large. When comparing these samples to those prepared by ball milling, a resemblance in the trends is evident. However, the optical trend of the manually ground samples after one month resembles the behavior of the ball-milled samples after just one week, highlighting the slower stabilization. For the STE emission, after one day, the trends of the blue shift and

red shift appeared more linear compared to the samples prepared by ball milling. After one week, the blue shift displayed more nonlinear pattern with minor bowing, followed by a sharp red shift for samples with higher bromide content. Only after one month did the optical trend develop a more pronounced curvature, with the blue shift showing nonlinear dependency and clear bowing. However, the red shift remained linear, and the energy differences between the mixed halide compounds were still minimal. This optical trend of samples prepared by manual grinding after one month closely resembled the optical trend observed in samples prepared by ball milling after just one day. The PL results suggests that the same processes occur in both series of samples; however, they progress much faster for the samples prepared by ball milling. Thus, the preparation method significantly influences the kinetics of the structural evolution and of the optical properties over time.

Table S1. Summary of Abs and PL extracted/calculated values of the manually grinded samples after one day

	Abs peak (eV)	Abs onset (eV)	FE peak maxima (eV)	STE peak maxima (eV)	STE peak intensity	FWHM (eV)	ΔE (Abs peak - STE peak) (eV)
**Cl**	3.9995	3.75	3.75758	2.36641	48143.15	0.984	1.63309
**0.1Br**	3.9739	3.405	3.46369	2.41245	424488.9	0.886	1.56145
**0.2Br**	3.8032	3.367	3.44444	2.43137	468760.6	0.9245	1.37183
**0.3Br**	3.5628	3.336	3.40659	2.46032	556386	0.955	1.10248
**0.4Br**	3.5526	3.318	3.36043	2.46521	503030.4	0.99	1.08739
**0.5Br**	3.5123	3.286	3.35135	2.44094	427420.8	1.008	1.07136
**0.6Br**	3.4925	3.294	3.3244	2.4313	262498.8	1.049	1.0612
**0.7Br**	3.4632	3.266	3.33333	2.422	277039.3	1.0341	1.0412
**0.8Br**	3.4345	3.238	3.29787	2.441	407237.9	1.0031	0.9935
**0.9Br**	3.425	3.239	3.25459	2.3892	372945.1	0.952	1.0358
**Br**	3.4155	3.232	3.2461	2.39382	409487.3	0.923	1.02168

Table S2. Summary of Abs and PL extracted/calculated values of the manually grinded samples after one week

	Abs peak (eV)	Abs onset (eV)	FE peak maxima (eV)	STE peak maxima (eV)	STE peak intensity	FWHM (eV)	ΔE (Abs peak - STE peak) (eV)
**Cl**	3.9612	3.72	3.73494	2.36641	101743.8	0.913	1.59359
**0.1Br**	3.9112	3.439	3.49296	2.41245	391905.2	0.8864	1.49755
**0.2Br**	3.8149	3.374	3.42541	2.43615	470351.6	0.918	1.37385
**0.3Br**	3.5323	3.346	3.42541	2.48996	1.37E+06	0.941	1.04004
**0.4Br**	3.5024	3.321	3.37875	2.51012	824969.1	0.991	0.98988
**0.5Br**	3.5223	3.304	3.36043	2.51521	554215.5	1.089	1.00479
**0.6Br**	3.4536	3.268	3.333	2.52546	438996.1	1.132	0.92454
**0.7Br**	3.4632	3.254	3.29787	2.5	369321.9	1.133	0.96
**0.8Br**	3.4345	3.222	3.28042	2.49497	332150.9	1.124	0.93503
**0.9Br**	3.425	3.218	3.25459	2.45059	29110.56	1.064	0.96941
**Br**	3.425	3.217	3.21244	2.45545	530505.9	0.936	0.96455

Table S3. Summary of Abs and PL extracted/calculated values of the manually grinded samples after one month

	**Abs peak (eV)**	**Abs onset (eV)**	**FE peak maxima (eV)**	**STE peak maxima (eV)**	**STE peak intensity**	**FWHM (eV)**	**ΔE (Abs peak - STE peak) (eV)**
**Cl**	3.9739	3.736	3.72372	2.37	320032.3	0.855	1.6039
**0.1Br**	3.9112	3.427	3.53276	2.43137	730777.6	0.877	1.47983
**0.2Br**	3.6359	3.348	3.44444	2.46521	726892.2	0.92	1.17069
**0.3Br**	3.5323	3.33	3.4349	2.48	902853.7	0.932	1.0523
**0.4Br**	3.4925	3.313	3.39726	2.50505	787042.5	0.996	0.98745
**0.5Br**	3.4729	3.283	3.37875	2.51521	473056.7	1.109	0.95769
**0.6Br**	3.4536	3.265	3.34232	2.51	764949.8	1.124	0.9436
**0.7Br**	3.4632	3.249	3.3244	2.49497	340219.2	1.152	0.96823
**0.8Br**	3.444	3.216	3.29787	2.485	312115.5	1.163	0.959
**0.9Br**	3.425	3.205	3.263	2.45545	256842	1.076	0.96955
**Br**	3.4345	3.207	3.263	2.445	435744.8	0.944	0.9895

Table S4. Summary of Abs and PL extracted/calculated values of the ball milling samples after one day

	Abs peak (eV)	Abs onset (eV)	FE peak maxima (eV)	STE peak maxima (eV)	STE peak intensity	FWHM (eV)	ΔE (Abs peak - STE peak) (eV)
**Cl**	3.9112	3.515	3.70149	2.37094	443404.9	0.808	1.54026
**0.1Br**	3.8504	3.3697	3.58382	2.41245	265347.2	0.879	1.43795
**0.2Br**	3.8032	3.3529	3.52273	2.455	441190.3	0.911	1.3482
**0.3Br**	3.652	3.3849	3.46369	2.46521	710138.7	0.937	1.18679
**0.4Br**	3.5424	3.3324	3.45404	2.47505	527919.1	0.986	1.06735
**0.5Br**	3.5628	3.2902	3.41598	2.48	530946.4	0.997	1.0828
**0.6Br**	3.5475	3.2744	3.36957	2.45059	456422.8	0.989	1.09691
**0.7Br**	3.4876	3.25207	3.3244	2.46032	320346.6	1.034	1.02728
**0.8Br**	3.4827	2.992	3.33333	2.44576	338701.9	1.029	1.03694
**0.9Br**	3.4632	3.2015	3.31551	2.4409	456637.7	0.993	1.0223
**Br**	3.4827	3.1668	3.29787	2.436	450746.5	0.935	1.0467

Table S5. Summary of Abs and PL extracted/calculated values of the ball milling samples after one week

	Abs peak (eV)	Abs onset (eV)	FE peak maxima (eV)	STE peak maxima (eV)	STE peak intensity	FWHM (eV)	ΔE (Abs peak - STE peak) (eV)
**Cl**	3.9236	3.68	3.71257	2.37094	414366.6	0.808	1.55266
**0.1Br**	3.8268	3.449	3.5942	2.42661	746237.6	0.847	1.40019
**0.2Br**	3.78	3.413	3.51275	2.47012	1.33E+06	0.896	1.30988
**0.3Br**	3.625	3.359	3.47339	2.5	1.33E+06	0.95	1.125
**0.4Br**	3.5834	3.337	3.44444	2.52546	1.22E+06	0.99	1.05794
**0.5Br**	3.5526	3.293	3.41598	2.54098	1.19E+06	1.06	1.01162
**0.6Br**	3.4925	3.278	3.37875	2.55144	1.03E+06	1.12	0.94106
**0.7Br**	3.5123	3.252	3.35135	2.52546	401676.9	1.174	0.98684
**0.8Br**	3.4729	3.241	3.31551	2.49497	372939.2	1.178	0.97793
**0.9Br**	3.4536	3.221	3.30667	2.48996	440600.9	1.13	0.96364
**Br**	3.4536	3.235	3.26316	2.44094	541636.6	0.0919	1.01266

Table S6. Summary of Abs and PL extracted/calculated values of the ball milling samples after one month

	**Abs peak (eV)**	**Abs onset (eV)**	**FE peak maxima (eV)**	**STE peak maxima (eV)**	**STE peak intensity**	**FWHM (eV)**	**ΔE (Abs peak - STE peak) (eV)**
**Cl**	3.936	3.7056	3.72372	2.37548	463558.1	0.808	1.56052
**0.1Br**	3.8989	3.525	3.5942	2.43137	1.06E+06	0.84	1.46753
**0.2Br**	3.8032	3.421	3.53276	2.47012	1.54E+06	0.896	1.33308
**0.3Br**	3.6359	3.358	3.47339	2.50505	1.39E+06	0.948	1.13085
**0.4Br**	3.5834	3.32	3.44444	2.53061	1.30E+06	1.002	1.05279
**0.5Br**	3.5526	3.309	3.39726	2.54098	1.51E+06	1.004	1.01162
**0.6Br**	3.5223	3.272	3.37875	2.55144	1.21E+06	1.0072	0.97086
**0.7Br**	3.5024	3.244	3.35135	2.5462	438421.5	1.14	0.9562
**0.8Br**	3.4632	3.227	3.33333	2.52033	401839.7	1.17	0.94287
**0.9Br**	3.4345	3.197	3.30667	2.5	563868.3	1.132	0.9345
**Br**	3.444	3.215	3.28912	2.46521	421818.1	0.955	0.97879

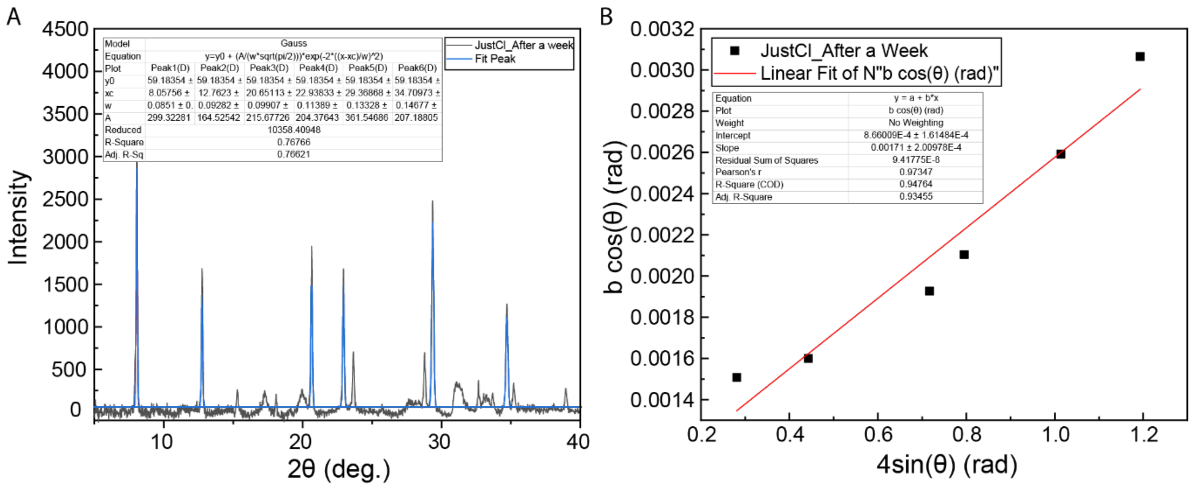

Figure S7. Determination of the microstrain value (slope), and crystal size (y-intercept) of the pure chloride sample prepared by ball milling, obtained by analyzing the pXRD peaks (A) and linear fitting using the W-H method (B).

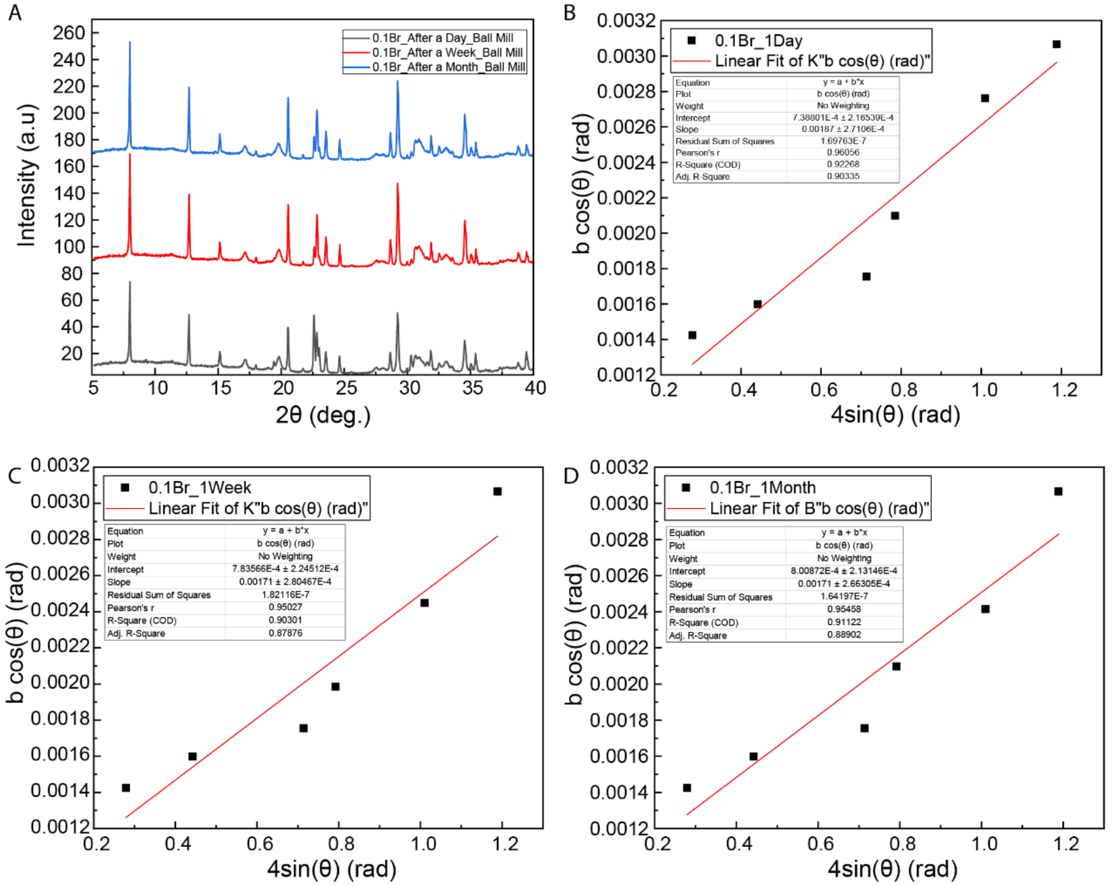

Figure S8. Determination of the microstrain value and crystal size of the (2,5-dmpz)Pb($Br_{0.1}Cl_{0.9}$)$_4$ prepared by ball milling over time. A. The measured pXRD patterns., B,C,D The liner fit of the samples after one day, one week, and one month, respectively.

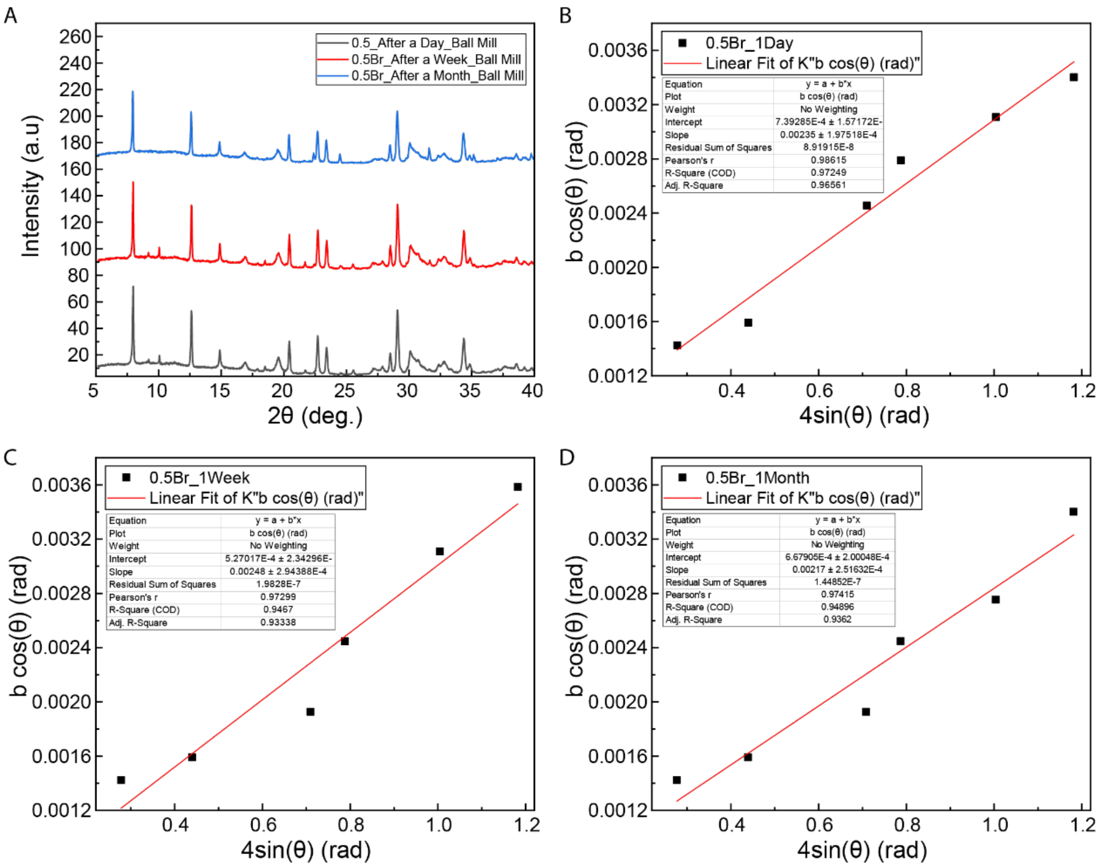

Figure S9. Determination of the microstrain value and crystal size of the (2,5-dmpz)Pb($Br_{0.5}Cl_{0.5}$)$_4$ prepared by ball milling over time. A. The measured pXRD patterns., B,C,D The liner fit of the samples after one day, one week, and one month, respectively.

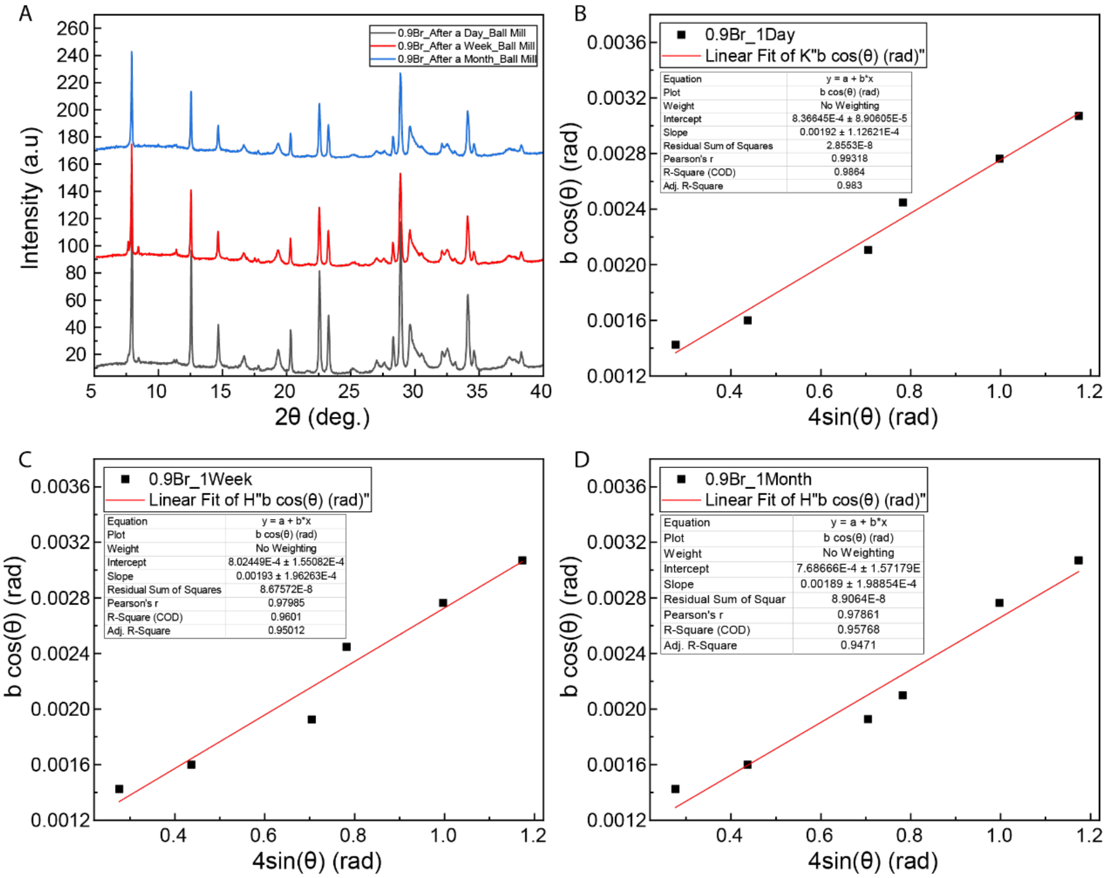

Figure S10. Determination of the microstrain value and crystal size of the (2,5-dmpz)Pb($Br_{0.9}Cl_{0.1}$)$_4$ prepared by ball milling over time. A. The measured pXRD patterns., B,C,D The liner fit of the samples after one day, one week, and one month, respectively.

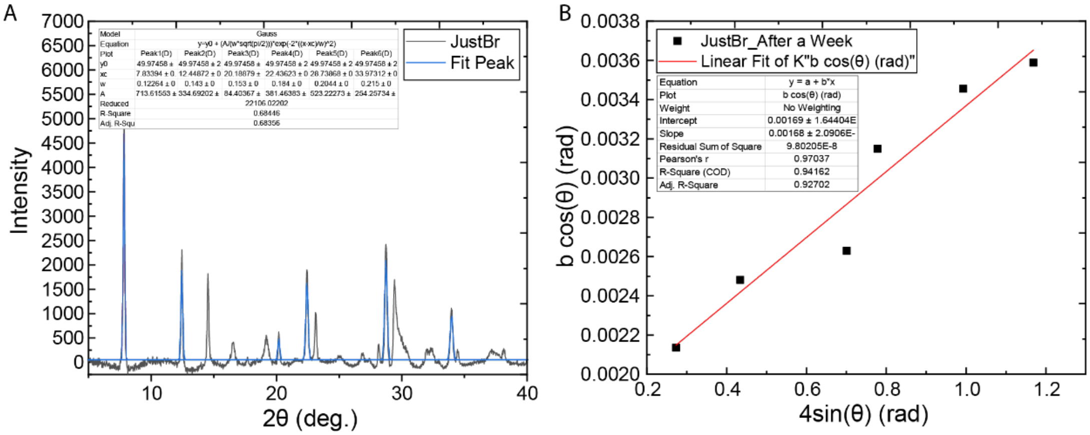

Figure S11. Determination of the microstrain value (slope), and crystal size (y-intercept) of the pure bromide sample prepared by ball milling, obtained by analyzing the pXRD peaks (A) and linear fitting using the W-H method (B).

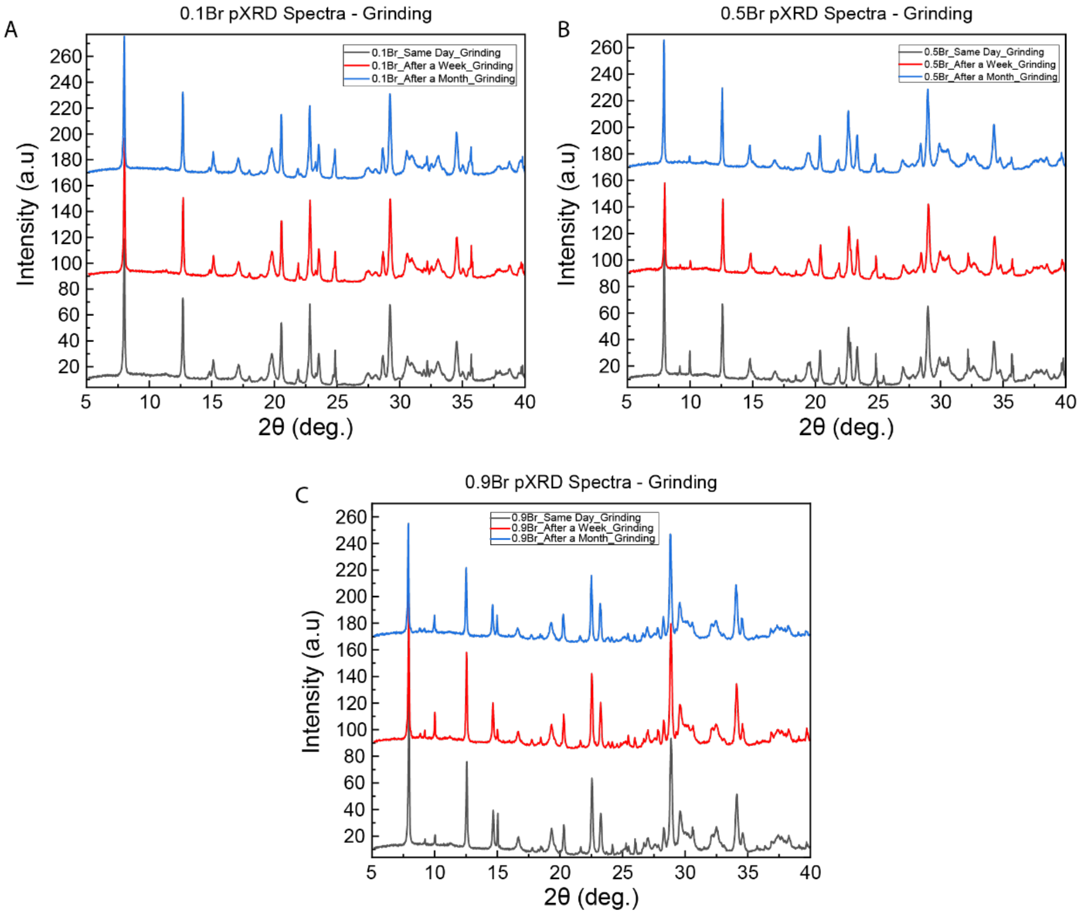

Figure S12. pXRD spectra of the manually grinded samples after one day, one week, and one month for samples of different compositions, 0.1 Br (A), 0.5 Br (B), and 0.9 Br (C).

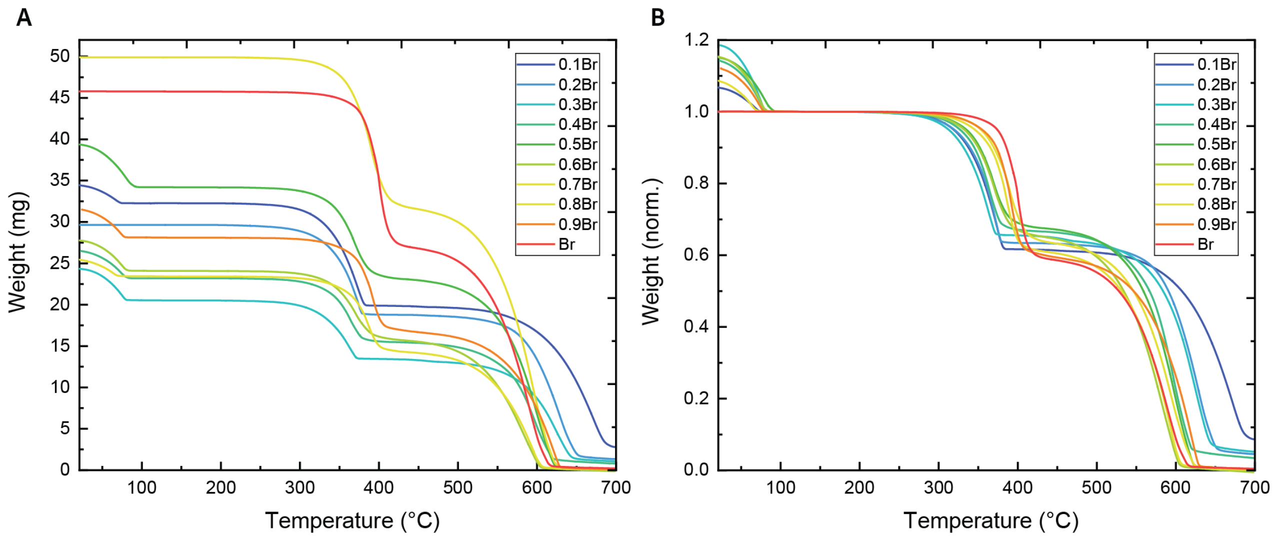

Figure S13. TGA measurements (A) and normalized TGA measurements (B) of (2,5-dmpz)Pb(Br_xCl_{1-x})$_4$ compounds prepared by ball milling. For some samples a mass loss up to 100 °C is observed, which is associated to evaporation of some residual humidity. The TGA spectra were normalized at higher temperature (200 °C) and the mass loss portion was calculated based on these spectra without the residual humidity.

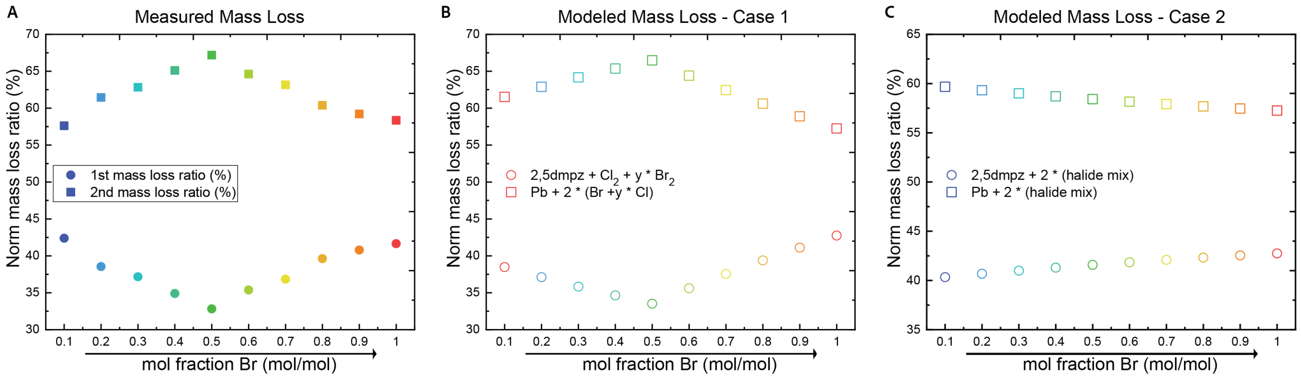

Figure S14. Mass loss of (2,5-dmpz)Pb(Br_xCl_{1-x})$_4$ compounds prepared by ball milling. A. The measured mass loss ratio for the 1st and 2nd events. B. Modeled mass loss ratio based on the hypothesis that Cl is leaving before the Br. C. Modeled mass loss ratio based on co-evaporation of the halides according to the compounds' composition. Clearly the model represented in B is much better fitted to the measured results.

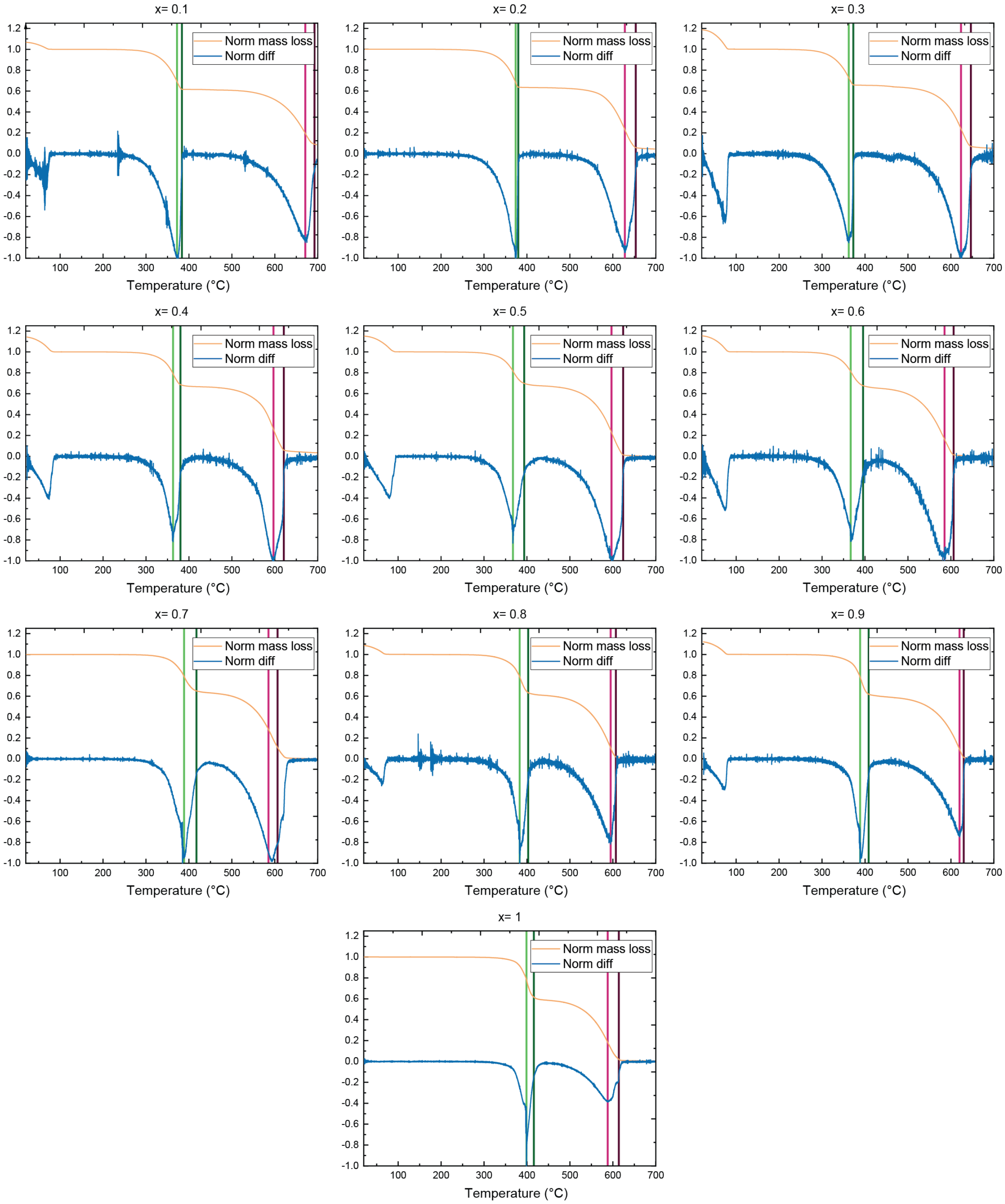

Figure S15. Normalized TGA and TGA derivative of (2,5-dmpz)Pb(Br_xCl_{1-x})$_4$ compounds prepared by ball milling. The decomposition temperatures where extracted based on the TGA derivatives minima (light green and pink lines) and based on the TGA spectra according to the decomposition event end (dark green and purple lines). Both methods yield in similar composition dependance. For further analysis we choose to use the TGA derivative minima which is free user bias.

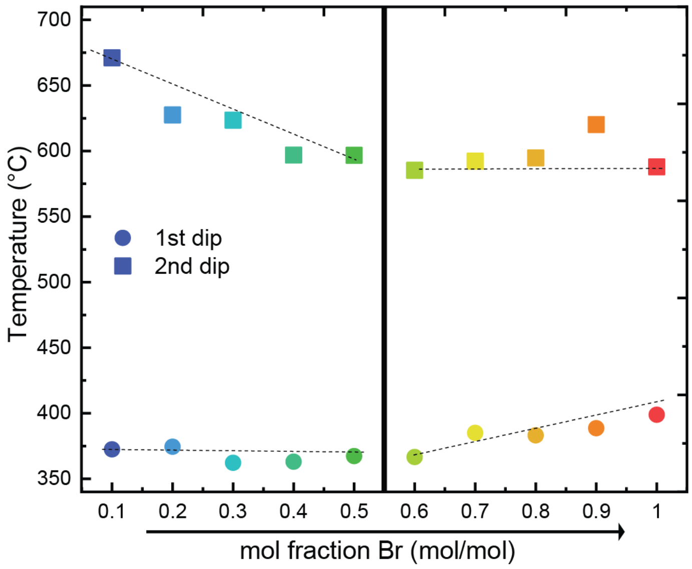

Figure S16. Mass loss temperatures for (2,5-dmpz)Pb(Br_xCl_{1-x})$_4$ compounds prepared by ball milling.

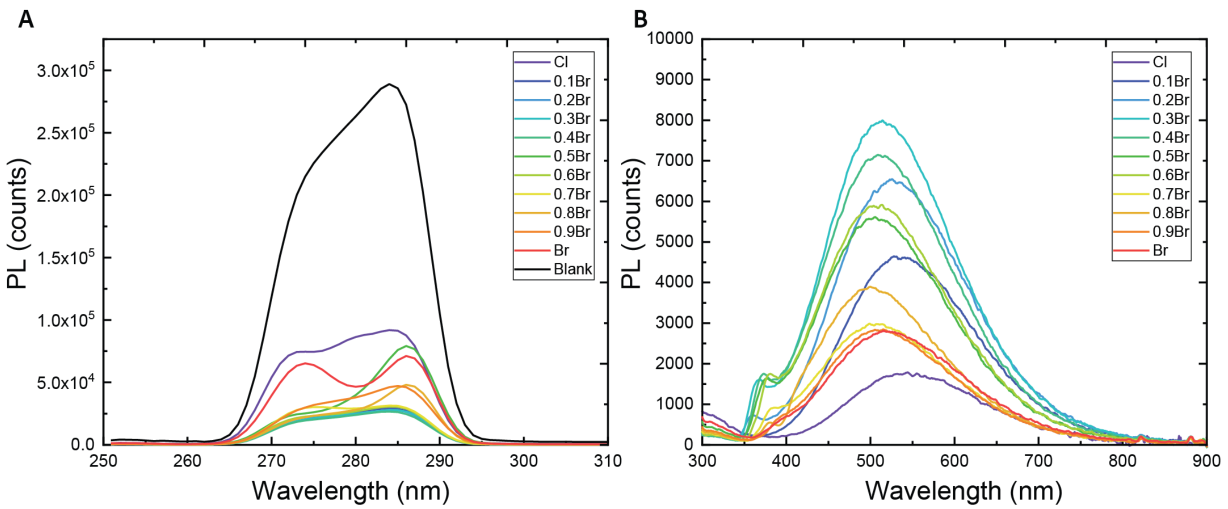

Figure S17. PLQY measurements of (2,5-dmpz)Pb(Br_xCl_{1-x})$_4$ compounds prepared by ball milling

Table S7. PLQY and optical properties of broad emitters with 1D structure

Compound	Connectivity motifs	PL CWL (nm)	PL FWHM (nm)	PLQY (%)	Ref.
**$C_4N_2H_{14}PbBr_4$**	ES dimers and CS wires	475	157	18-20	55
**(2,6dmpz)$PbBr_4$**	Mixed - ES dimers forming CS wires, and CS monomers	585	~160	12	64
**(hep)$PbBr_3$**	FS	674	~200	<1	64
**(hex)$PbBr_3$**	FS	681	~200	<1	64
**$(C_5H_{13}N_2)PbCl_4\cdot H_2O$**	ES dimers and ES wires	617	~210	1	56
**(2,5dmpz)$PbBr_4$**	ES dimers and CS wires	520	~200	45	50
**(2,5dmpz)$PbCl_4$**	ES dimers and CS wires	530	~200	7.5	83
**$(H_2O)(C_6H_8N_3)_2Pb_2Br_{10}$**	CS	580	~200	9	43
**[DTHPE]$_{0.5}PbCl_3$**	FS	458	185	6.99	51
**[DMTHP]$PbCl_3$**	FS	524	184	5.29	51
**[DBN]$PbCl_3$**	FS	460	158	1.49	51
**[DAPr-pipz]$PbCl_4\cdot H_2O$**	CS	532	173	1.64	57
**$[C_4N_2H_{12}]_3[PbBr_5]_2\cdot$4DMSO**	CS	560	~200	60	58
**(dbod)Pb_2Br_6**	CS, ES, FS	648	244	N.A	59
**[H_2BPP]Pb_2Br_6**	FS dimers and ES wires	524	105	8.1	52
**[H_2BPP]Pb_2Cl_6**	FS dimers and ES wires	538	112	4.84	52
**$Pb_2Br_9(C_5H_7N_5)_4PbBr_5\cdot 2H_2O$**	Mixed - CS monomers and CS Corrugated dimers	605	209	42.2	60
**(2,5dmpz)$PbCl_4$**	ES dimers and CS wires	522	183	11	This work
**(2,5dmpz)$Pb(Cl_{0.9}Br_{0.1})_4$**	ES dimers and CS wires	510	182	20.3	This work
**(2,5dmpz)$Pb(Cl_{0.8}Br_{0.2})_4$**	ES dimers and CS wires	502	188	28.6	This work
**(2,5dmpz)$Pb(Cl_{0.7}Br_{0.3})_4$**	ES dimers and CS wires	495	194	35	This work
**(2,5dmpz)$Pb(Cl_{0.6}Br_{0.4})_4$**	ES dimers and CS wires	490	202	31.1	This work
**(2,5dmpz)$Pb(Cl_{0.5}Br_{0.5})_4$**	ES dimers and CS wires	488	201	27.9	This work
**(2,5dmpz)$Pb(Cl_{0.4}Br_{0.6})_4$**	ES dimers and CS wires	486	200	26	This work
**(2,5dmpz)$Pb(Cl_{0.3}Br_{0.7})_4$**	ES dimers and CS wires	487	229	13	This work
**(2,5dmpz)$Pb(Cl_{0.2}Br_{0.8})_4$**	ES dimers and CS wires	492	241	16.6	This work
**(2,5dmpz)$Pb(Cl_{0.1}Br_{0.9})_4$**	ES dimers and CS wires	496	236	13.1	This work
**(2,5dmpz)$PbBr_4$**	ES dimers and CS wires	503	202	15.3	This work

Refernces are associated with main text. Connectivity motifs – CS – corner sharing, ES – edge sharing, FS – face sharing. PL CWL – central wavelnegth of the STE emission. PL FWHM – full width half maximum of the STE emission peak.
